# The R-loop grammar predicts R-loop formation under different topological constraints

**DOI:** 10.1371/journal.pcbi.1013376

**Published:** 2025-08-29

**Authors:** Margherita Maria Ferrari, Svetlana Poznanović, Manda Riehl, Jacob Lusk, Stella Hartono, Georgina Gonzalez-Isunza, Frédéric Chédin, Mariel Vázquez, Nataša Jonoska

**Affiliations:** 1 Department of Mathematics, University of Manitoba, Winnipeg, Manitoba, Canada; 2 Department of Mathematical and Statistical Sciences, Clemson University, Clemson, South Carolina, United States of America; 3 Department of Mathematics, Rose-Hulman Institute of Technology, Terre Haute, Indiana, United States of America; 4 Department of Microbiology and Molecular Genetics, University of California Davis, Davis, California, United States of America; 5 Department of Molecular and Cellular Biology, University of California Davis, Davis, California, United States of America; 6 Department of Mathematics, University of California Davis, Davis, California, United States of America; 7 Department of Mathematics and Statistics, University of South Florida, Tampa, Florida, United States of America; University of Missouri, UNITED STATES OF AMERICA

## Abstract

R-loops are transient three-stranded nucleic acids that form during transcription when the nascent RNA hybridizes with the template DNA, freeing the non-template strand of the DNA. There is growing evidence that R-loops play important roles in physiological processes such as the regulation of gene expression, and that they contribute to chromosomal instability and disease. It is known that R-loop formation is influenced by both the sequence and the topology of the DNA substrate, but many questions remain about how R-loops form and the three-dimensional structures that they adopt. Here we represent an R-loop as a word in a formal grammar, the *R-loop grammar*. We use the R-loop grammar to predict R-loop formation. We train the R-loop grammar on experimental data obtained by single-molecule R-loop footprinting and sequencing (SMRF-seq). Despite not explicitly encoding topological information, the R-loop grammar accurately predicts R-loop formation on plasmids with varying starting topologies and outperforms previous methods in R-loop prediction.

## Author summary

R-loops are transient three-stranded helical structures that form when newly synthesized RNA binds back to the DNA template strand during transcription, displacing the non-template strand. R-loops play important roles in regulating gene expression and are associated with genome instability linked to various diseases. While it is known that both the DNA sequence and topological state of the substrate affect R-loop formation, the precise rules behind how and where they form remain poorly understood. In our work, we develop a new mathematical model based on formal language theory to model R-loops. We define a predictive formal grammar model of R-loop formation, called the *R-loop grammar*, by first creating a symbolic language with specific production rules. We represent each R-loop as a word in this language. We train the R-loop grammar using high-resolution experimental data obtained by a single-molecule technique that maps R-loops formed during transcription. These data include a large set of R-loops formed on two different plasmids of varying DNA topologies. Despite not directly encoding topology, the R-loop grammar distills its effects, accurately predicts R-loop formation, and outperforms prior methods. This approach offers new insights into the relationship between DNA sequence, topology, R-loop formation and structure.

## Introduction

R-loops are three-stranded structures composed of an RNA:DNA duplex and a single-strand of DNA. Initially discovered in bacteria, R-loops constitute 3-5% of the genome of yeasts, plants, and mammals [1–6] and are at least one order of magnitude longer than other non-B DNA multi-stranded nucleic acid structures [7,8].

**Fig 1:**
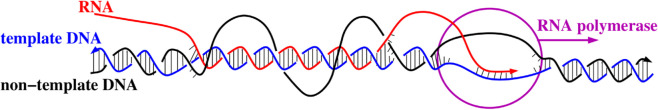
Co-transcriptional R-loops. The RNA polymerase mediates transcription of DNA into RNA. A co-transcriptional R-loop forms behind the polymerase when the RNA transcript invades the double-stranded DNA (dsDNA) and hybridizes with the template DNA strand. The template and non-template DNA strands are shown in blue and black, respectively. The red strand represents the RNA transcript. In the R-loop, the non-template DNA strand is unpaired and free to wrap around the RNA:DNA duplex. The $3^{\prime }$-ends are indicated by an arrowhead.

**Fig 2:**
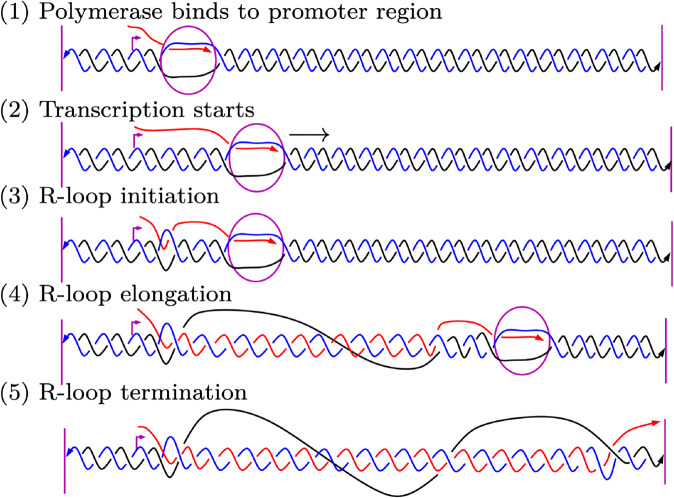
Stages of R-loop formation. (1) The RNA polymerase binds to the promoter sequence (purple arrow). (2) Transcription starts. The polymerase moves from left to right and generates the RNA transcript (in red) in the $5^{\prime }$ to $3^{\prime }$ direction. (3) R-loop initiation: the nascent RNA invades the dsDNA and hybridizes to the DNA template strand (in blue). (4-5) R-loop elongation and eventual termination.

R-loops form co-transcriptionally when the nascent RNA invades the DNA duplex and the RNA hybridizes with the template DNA strand [9]. The unpaired non-template DNA strand is free to wrap around the hybrid duplex (Fig 1) or to fold upon itself into a secondary structure. R-loops arise through a dynamic process that begins with DNA duplex invasion by the nascent RNA behind the advancing RNA polymerase (*initiation phase*). Once an R-loop has been seeded, it can extend dynamically during transcription (*elongation phase*). Having reached a point where the structure can no longer grow, the R-loop terminates (*termination phase*). Termination is followed by an equilibration process, where the exact boundaries of the structure may shift through branch migration [10]. Eventually, the R-loop dissociates and the B-form DNA duplex is restored. Fig 2 illustrates the different stages of R-loop formation.

Organisms have evolved complex pathways that regulate R-loop levels [11]. Genome mapping studies indicate that R-loops do not form randomly [9,12]. DNA sequence analysis, biochemical experiments and statistical mechanical modeling suggest that both the DNA sequence and the topology play key roles in promoting and controlling R-loop formation [9,13]. The fundamental forces that drive R-loop initiation, elongation and termination are poorly understood.

In this work we develop a new mathematical model based on formal language theory to model R-loops. In formal language theory, a grammar is a set of production rules that generate strings in a formal language. Applications of formal grammars can be found in a wide range of areas such as theoretical computer science, theoretical linguistics, and molecular biology. In molecular biology, applications include modeling regulation of gene expression [14], gene structure prediction [15], sequence analysis [16] and RNA secondary structure prediction [17].

We introduce the *R-loop grammar*, a predictive formal grammar model of R-loops that advances our understanding of the structure, formation and biological function of R-loops. We train and test the R-loop grammar on experimental data obtained by single-molecule RNA footprinting and sequencing (SMRF-seq) [8,18]. We use the data to define the syntax of the R-loop language and to obtain the probabilities of the production rules, thus allowing us to write each R-loop as a word in this language. The grammar model predicts the probability that an R-loop will form along a given DNA segment, the location of the R-loop and its basic 3-dimensional (3D) structure.

**Fig 3:**
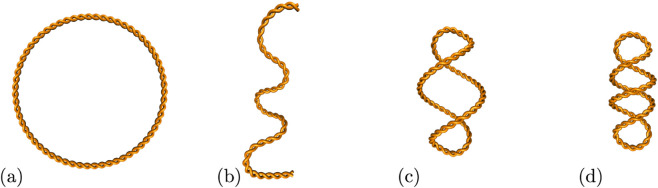
Plasmid topologies considered in this study. (a) Open circle. The open circle represents a relaxed plasmid; (b) Linear. The curve with two free ends represents the linearized version of the plasmid. (c) and (d) Supercoiled. B-DNA has a helical pitch of 10.5 base pairs (bp) per turn. In living organisms DNA is slightly underwound. In order to preserve a constant helical pitch, underwound DNA coils upon itself in a right-handed fashion, thus producing negative supercoils. We assign the sign of the coiling based on a standard sign convention. (c) Negatively supercoiled plasmid. (d) Hyper-negatively supercoiled plasmid, where the level of supercoiling is twice that of the supercoiled plasmid. The supercoiled plasmids in this study have the native supercoiling from *Escherichia coli* (supercoiling density ~−0.07) and the hyper-negatively supercoiled plasmids (supercoiling density ~−0.14) are produced by gyrase, a bacterial type II topoisomerase. Further information on DNA topology can be found in [19].

We took advantage of a SMRF-seq R-loop mapping dataset generated after *in vitro* transcription of two plasmids, pFC53 and pFC8 [8,13,18]. In contrast with other methods that output population averages, SMRF-seq provides high coverage, strand-specific information about R-loops at nucleotide resolution on individual DNA molecules. Thus, SMRF-seq allows researchers to capture the positions and lengths of single R-loops. The data include detailed information on R-loop formation under three topological conditions: linear, negatively supercoiled and hyper-negatively supercoiled. See Fig 3 for a description of the different DNA topologies.

**Fig 4:**
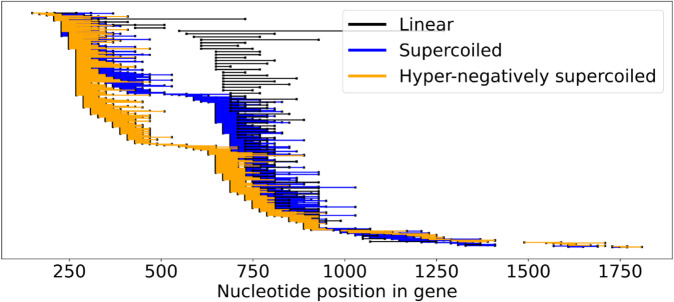
Experimental data for pFC53. R-loop locations with starting topology: linear (black); supercoiled (blue); and hyper-negatively supercoiled (orange). The *x*-axis indicates the nucleotide position of the gene starting at 0 rounded to the nearest $20^{\text{th}}$ nucleotide. Each horizontal line segment corresponds to one experimentally detected R-loop. The R-loops have been sorted by the starting nucleotide (*x*-axis). Each data set is uniformly spread vertically (79 R-loops for linear, 612 for supercoiled and 408 for hyper-negatively supercoiled), so that proportional differences in R-loop initiation under the three conditions can be observed independent of the number of experimental R-loops observed.

Our experimental data consist of a total of 2,363 single-molecule reads with one R-loop each (see Fig 4, S1 Fig and Materials and methods section). Fig 4 illustrates the data for pFC53 and the influence of the plasmid topology on R-loop formation; the corresponding figure for pFC8 is S1 Fig in the Supporting Information. Note that R-loops cluster in two regions for all substrate topologies and that R-loop initiation shifts to the left as the supercoiling levels increase. As observed in [13], the majority of the R-loops in the hyper-negatively supercoiled plasmid appear closer to the transcription start; this is not the case for the other two topologies (Fig 4).

Here we show that the R-loop grammar model distills the effect of DNA topology on R-loop formation. Unlike the R-looper prediction method [13], our model does not explicitly include parameters to specify the topology of the substrate. Instead, the R-loop grammar learns about these topological constraints from the data. A key finding is that the substrate topology affects the probability of the production rules. Consequently, the R-loop grammar accurately predicts the probability of R-loop formation.

## The R-loop grammar: A model for R-loop formation

### Formal grammars and R-loops

A formal grammar consists of a finite set of symbols partitioned into *variables V* and *terminals* Σ, and a finite set of *production rules* {u→v}. When applying the rule u→v on a word *xuy*, the subword *u* is substituted by the subword *v* yielding a word *xvy*. A word derived by the grammar is obtained by a consecutive application of rules starting from *S*, a non-terminal symbol designated as a *starting symbol*. The language generated by the grammar consists of all words comprised of terminal symbols that can be derived by the rules starting from *S* [20–22].

**Fig 5:**
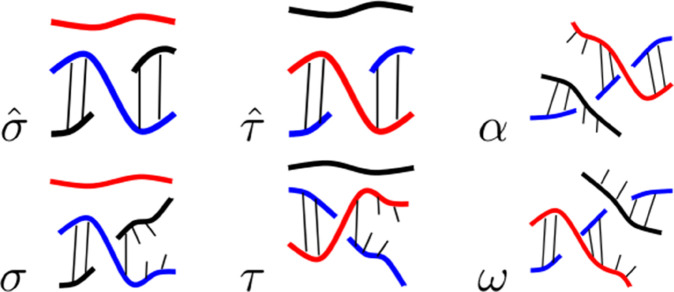
R-loop symbols. Basic 3-strand structures found in an R-loop and their associated symbols in the R-loop grammar. We indicate less stable configurations *σ* and *τ* by breakage in the hydrogen bonds. This representation should not be taken as literal breakage of all bonds in that vicinity, but rather as an indication that this region is unstable and prone to opening of the helix. The color coding is as in Fig 1.

**Fig 6:**
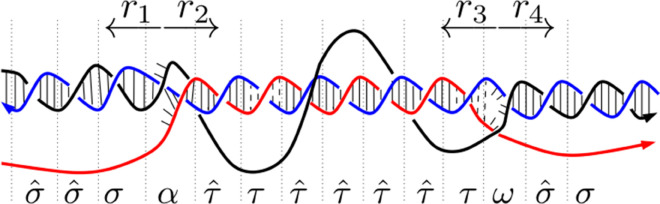
An example of an R-loop grammar word. The figure illustrates an R-loop associated with the word $\hat{\sigma }\hat{\sigma }\sigma \alpha \hat{\tau }\tau \hat{\tau }\hat{\tau }\hat{\tau }\hat{\tau }\tau \omega \hat{\sigma }\sigma$. The colors are as in Fig 1. For simplicity, we omit the broken hydrogen bonds for *σ* and *τ*. We indicate stability with the symbols under the diagram.

We define the *R-loop grammar* as a formal grammar whose terminal symbols correspond to the basic structures of an R-loop. The three-strand sections *α* and *ω* correspond to those regions of branch migration that mark the initiation (RNA invasion) and termination (RNA dissociation) of the R-loop, respectively. The symbols *σ* and $\hat{\sigma }$ represent short DNA:DNA hybrids with a free RNA strand (the RNA transcript). The symbols *τ* and $\hat{\tau }$ represent RNA:DNA hybrids with a free DNA strand (the non-template strand). The ‘ ^^^ ’ indicates a more stable configuration, i.e. a configuration that is not prone to changing state. Therefore, $\hat{\sigma }$ denotes a structure unlikely to transition from a DNA duplex to an RNA:DNA hybrid, and $\hat{\tau }$ denotes a structure unlikely to transition from an RNA:DNA hybrid back into the DNA duplex. Fig 5 illustrates the main terminal symbols in the R-loop grammar. Fig 6 shows a word generated by the R-loop grammar and its corresponding R-loop structure. Note that if the sequence stability weakens within an R-loop, a less stable RNA:DNA duplex (indicated by *τ*) may follow after an initial string of one or more $\hat{\tau }$’s. Intuitively, one or more consecutive *τ*’s may lead to an R-loop termination region.

To determine probability assignments of the terminal symbols in the R-loop grammar and the application of each production rule, we first extract a training set from two thirds of the experimental data. We use the remaining third of the data for testing the method. The application of each production rule depends on a probability distribution generated from the data.

We apply this approach to the SMRF-seq data for plasmids pFC53 and pFC8 with three different plasmid topologies. The reader can find details of the method in section General approach and in section Materials and methods.

### Symbol assignment and R-loop production rules

We use SMRF-seq data to determine the probabilities for the R-loop grammar production rules (all the experimental data are available on GitHub [23] and Zenodo [24]). To each block of *k* consecutive nucleotides (*k*-mer) in the gene sequence, we assign a terminal symbol according to the probability that it is contained in an R-loop. The words generated by the grammar correspond to R-loops (Fig 6).

Occasionally the basic terminal symbols cannot be unequivocally assigned to a *k*-mer based on the available experimental data. In those instances, we expand the set of terminal symbols to accommodate the corresponding *k*-mers. The symbol *δ* (respectively, *β*) represents ambiguous *k*-mers that, according to the statistical analysis of the training data, could be associated with both *σ* and $\hat{\sigma }$ (respectively, *τ* and $\hat{\tau }$). The *k*-mers outside (respectively, within) an R-loop for which the statistical analysis does not provide enough information are indicated with *γ* (respectively, *ρ*). Moreover, to account for the fact that the experimental assignment of initiation and termination of each R-loop is not precise [8], the terminals *α* and *ω* corresponds to a segment of length 0,…,k−1. Hence, the R-loop grammar alphabet consists of 10 letters ($\alpha ,\omega ,\sigma ,\hat{\sigma },\tau ,\hat{\tau },\gamma ,\rho ,\beta ,\delta )$ and is independent of the *k*-mer size *k*.

As is common in formal grammars, the non-terminal symbols are written with capital symbols, and rules X→Y and X→Z with the same left side are written as X→Y∣Z. We define the following rules:


$$\text{A. start rule}\;\;S\to \sigma S\;|\;\hat{\sigma }S\;|\;\gamma S\;|\;\delta S\;|\sigma \alpha R\;|\;\hat{\sigma }\alpha R\;|\;\gamma \alpha R\;|\;\delta \alpha R\;\;\text{B.RNA:DNAduplex}\;\;R\to \tau R\;|\;\hat{\tau }R\;|\;\rho R\;|\;\beta R\;|\;\tau \omega Q\;|\;\hat{\tau }\omega Q\;|\;\rho \omega Q\;|\;\beta \omega Q\;\;\text{C.DNA:DNAduplex}\;\;Q\to \sigma Q\;|\;\hat{\sigma }Q\;|\;\gamma Q\;|\;\delta Q\;|\;\sigma \;|\;\hat{\sigma }\;|\;\gamma \;|\;\delta \;$$

The sequence analysis in the Materials and methods section describes a way to map each *k*-mer to a terminal symbol. Such assignments allow each R-loop to be represented as a word over the symbols in this grammar. The word can be obtained by starting with the symbol *S* and applying a unique sequence of rules. For example, by applying the rules S→σαR, $R\to \hat{\tau }\omega Q$, and Q→σ in succession one obtains the word $\sigma \alpha \hat{\tau }\omega \sigma$. We explain the probability assignments for each production rule in the Obtaining a model section and S1 Text. The probability assigned to each R-loop is computed as the product of the probabilities of the corresponding production rules. For example, the probability $\mathbb{P}(\sigma \alpha \hat{\tau }\omega \sigma )$ of the R-loop described by the word $\sigma \alpha \hat{\tau }\omega \sigma$ is defined as

$$\mathbb{P}(\sigma \alpha \hat{\tau }\omega \sigma )=\mathbb{P}(S\to \sigma \alpha R)\mathbb{P}(R\to \hat{\tau }\omega Q)\mathbb{P}(Q\to \sigma ).$$ (1)

### General approach

One goal when analyzing experimental R-loop data on a given gene sequence is to identify genomic patterns specific to the initiation, elongation, and termination of R-loops. For each R-loop in the training set, we identify four regions of interest, *r*_1_ and *r*_2_ immediately upstream and downstream of the R-loop initiation, as well as *r*_3_ and *r*_4_ immediately upstream and downstream of the R-loop termination (Fig 6). For each *i*, we consider all of the *k*-mers that appear in region *r*_*i*_ and assign weights according to the relative frequency of the respective *k*-mer (see section Selecting the most relevant *k-mers*). We compile a list of *k*-mers (across all R-loops) in each region, and use the weights to associate a terminal grammar symbol to each *k*-mer, thus forming a dictionary that is then used to generate a grammar model.

To generate a grammar model–a probability assignment for each grammar rule–we translate the R-loops from a training set into words over the grammar symbols. Then we reverse-engineer the sequences of production rules that generate the words and assign probabilities to the rules according to the frequencies of each rule application (see section S1 Text). To make predictions, we first use the grammar to generate all possible R-loop words for a given dataset. The probability $\bar{\mathbb{P}}(w)$ of a word *w* is proportional to the product of the probabilities of the sequence of rules that generate *w* (e.g. Eq 1). We compute the probability of each nucleotide being in an R-loop by summing the probabilities of the words where this event occurs. For example, let *q*_*i*_(*w*) be 1 if the *i*-th nucleotide is in the R-loop represented by *w*, and 0 otherwise. The probability $\bar{q_{i}}$ that the *i*-th nucleotide is in an R-loop is

$$\bar{q_{i}}=\sum_{w}q_{i}(w)\bar{\mathbb{P}}(w),$$ (2)

where the sum is taken over all possible R-loop words in the the dataset.

For each plasmid and each starting topology, we take a portion of the experimental data as a *holdout set*. We take a random 10% of the remaining data, use it for training and to generate a grammar model. We repeat this process 30 times to generate an ensemble of 30 grammar models (see sections Results, Materials and methods and S2 Fig). In the Results section, we show the average probability that each nucleotide is in an R-loop obtained from the ensemble of models.

## Results

For each plasmid, we analyze three datasets that differ in the DNA topology before transcription: *linear*, where circular plasmids are linearized before transcription; *supercoiled*, where the plasmids have the native supercoiling from bacteria, i.e., a supercoiling density of ~−0.07; and *hyper-negatively supercoiled*, where the plasmids are treated with gyrase before transcription to double the supercoiling density to ~−0.14 [19] (see Fig 3).

**Fig 7:**
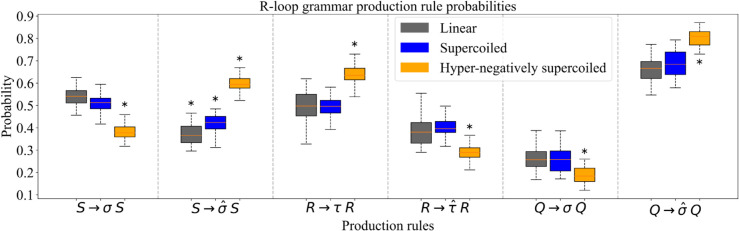
Production rule probabilities. The boxplots illustrate the changes in the probabilities for the six main production rules that relate to the stability of the structure before, within, and after an R-loop, as the topology from the substrate changes from linear to hyper-negatively supercoiled. The probabilities are obtained for grammar models defined for the union of training sets, with parameters *k* = 4 and *p* = 13. The mid-line of each box is the median, with the first and third quartiles indicated by the box frames. The whiskers represent the largest point not more than 1.5 interquartile range (IQR) beyond the box frame. An asterisk ^(*)^ indicates that the difference is significant against the results from the other two topologies (p≤0.006). The significance of these probability changes are obtained with Bonferroni adjusted *p*-values ≤0.006 according to the pairwise T-test. See S1 Table for precise values.

**Fig 8:**
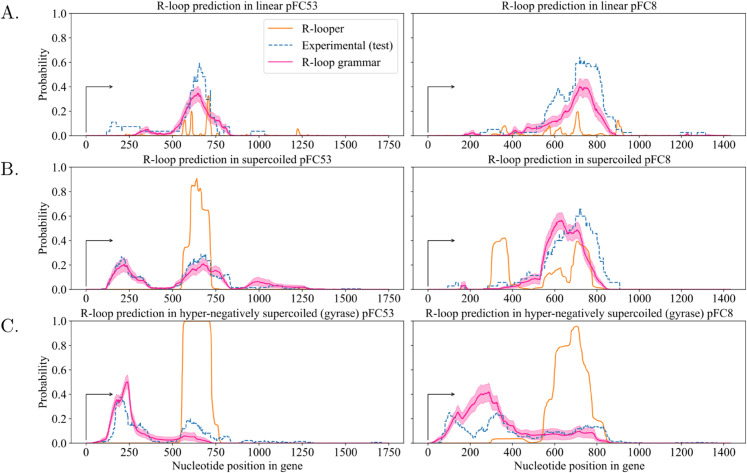
R-loop grammar predictions compared to experiments and to R-looper. The figure shows the R-loop grammar predictions obtained with dictionaries for k=4,p=13. The dotted blue line indicates the experimental probability that a nucleotide in the plasmid with the respective topology is in an R-loop. In pink (resp. orange) we show the R-loop probabilities computed using the R-loop grammar model (resp. R-looper). The pink shaded area represents the standard error of the mean. For each plasmid, we compute the experimental probabilities by taking the number of R-loops that contain a particular nucleotide (x-axis), divided by the total number of R-loops in the holdout set. The black arrow along the x-axis indicates the start of transcription; nucleotides are enumerated from that position. The substrate topology is indicated in each graph: linear (top row); supercoiled (middle row); hyper-negatively supercoiled (bottom row).

We generate predictions using R-loop grammars obtained using a stochastic and a deterministic method. In this section we discuss results from the stochastic method (Figs 7 and 8, see also S3 Fig). The predictions with the deterministic method showed negligible differences (S4 Fig).

### Plasmid topology drives the probability of the production rules

Fig 7 shows the probability assignments for the production rules associated with symbols $\sigma ,\hat{\sigma },\tau ,\hat{\tau }$ averaged over an ensemble of 30 grammar models. The probabilities of the other productions rules are included in S5 Fig, panel (a). A higher probability for a given rule implies that the training set contains a larger number of *k*-mers associated with that symbol. When a *k*-mer repeats, its multiplicity is taken into account. Note that the production rule probabilities change significantly with plasmid topology, which is consistent with the premise in [13].

As the supercoiling level increases towards hyper-negatively supercoiled, the probability of a stable DNA duplex ($\hat{\sigma }$) outside the R-loop also increases (rules $S\to \hat{\sigma }S$ and $Q\to \hat{\sigma }Q$). This suggests that the *k*-mers outside the R-loop are well determined. Once an R-loop starts, the pattern is reversed and the probability of elongating a stable R-loop decreases as supercoiling levels increase (rule $R\to \hat{\tau }R$). We validated these observations using Kendall’s Tau correlation coefficient (S2 Table). Recall that when compared with linear and supercoiled plasmids, a significantly higher number of R-loops in hyper-negatively supercoiled plasmids appear closer to the transcription start (Fig 4). Because our dictionary assignment is focused on the regions at the start and the end of the R-loops, in hyper-negatively supercoiled plasmids a larger number of *k*-mers are spread throughout the R-loop containing regions (e.g., region between ~250nt and ~1250nt for pFC53, see Fig 4). This spread of the R-loop starting points in hyper-negatively supercoiled cases implies that *k*-mers within those R-loops have a somewhat weaker association with R-loops. Hence, rule R→τR occurs with a much higher probability than $R\to \hat{\tau }R$. If we instead focus on linear and supercoiled plasmids, we observe that *k*-mers are mostly concentrated around the peak of the R-loop clusters (~650nt to ~1250nt). Accordingly, the difference between probabilities R→τR and $R\to \hat{\tau }R$ is much smaller.

After an R-loop terminates, the probability of transitioning to a stable DNA duplex is high for all topologies. We observe the same trends in probabilities upon training the grammar on the data from each plasmid separately (S5 Fig(b) and S5 Fig(c)), as well as for the deterministic symbol assignment (S5 Fig(d)).

### The R-loop grammar accurately predicts R-loop formation for different topologies

**Fig 9:**
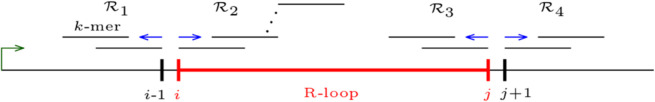
k-mer parsing. Sliding-window approach for extracting *k*-mers around the initiation and termination sites of an R-loop [*i*,*j*] in a training set ${𝒯}_{P}$.

The R-loop grammar model has two adjustable parameters, the tuple size *k* that is used for the dictionary and for the terminal symbol assignments, and the padding length *p* that is used to determine the size of regions $r_{1},\ldots ,r_{4}$ (see section *k-mer extraction*). Due to experimental sensitivity, the initiation of the R-loop may vary up to 15 nucleotides from the location observed through SMRF-seq [8]. To account for this, we focused on padding parameters *p*=7,13 (see S3 Table). The *k*-mer plus the padding correspond approximately to one (*p* = 7), or one and a half (*p* = 13) turns of an A-DNA double-helix (~11bp). RNA:DNA hybrids are believed to have the same helical pitch as A-DNA. Furthermore, we assume that the *k*-mers in the vicinity of the experimental R-loop start/end locations are critical for accurate prediction. We add the padding (nucleotide segments) before/after the R-loop start/end, thus defining the *k*-mers in regions *r*_1_ to *r*_4_ (see Fig 9).

In order to rigorously assess the best choice of parameters (*k*, *p*), for each topology we use the R-loop grammar to predict the probability of R-loop formation on each plasmid as detailed below (Fig 8). We then use 3-fold cross-validation to evaluate the model predictions for all (*k*, *p*) with k=3,4,5 and p=7,13 (find details in section Dictionary: Grammar symbol assignment for the set 𝒯). For each topology, we select the pair (*k*, *p*) that produces the lowest average root mean square deviation (RMSD) and the highest average Pearson correlation coefficient computed from the three validation sets. We found *k* = 4 and *p* = 13 to be the optimal parameters (see S3 Table).

It is worth noting that when *k* = 3, the symbol assignment in the regions *r*_1_ to *r*_4_ (see Fig 6 and section Materials and methods) exhausts all 64 possible 3-mers and the probabilities for production rules going to *γ* or *ρ* are 0, resulting in an oversimplified grammar model. When *k* = 5, the sequences of the two plasmids provide insufficient information, leaving between 39.1% and 58.1% of the 5-mers (p=7,13) assigned to an indeterminate symbol *γ* or *ρ* (S4 Table and S5 Table). The choice *k* = 4 and *p* = 13 is optimal as it provides information for 86.6% to 95.2% of all possible *k*-mers.

We generate the model predictions as follows. First, for each plasmid and topology, we randomly select one-third of the experimental data to serve as a holdout set for testing. The remaining two-thirds constitute the full training dataset. We produce an ensemble of 30 grammar models by training each model on a distinct 10% subsample (without replacement) drawn from the full training dataset (see section S1 Text). Hereafter we refer to this 10% of the data as the *training set* for the corresponding grammar model (see section Training and holdout set). Next we use the union of training sets for the two plasmids to create a *k*-mer dictionary. We reverse engineer the rules used from the R-loop grammar to define a probability of a given R-loop (as in Eq 1). Finally, we compute the probability that each nucleotide in the gene region is inside an R-loop using (Eq 2).

The final predictions assigning the probability that each nucleotide is within an R-loop are the average probabilities taken over the ensemble of 30 models (Fig 8) [25]. We tested the stability of the ensemble of predictions using 3-fold cross-validation [26] (see section Materials and methods and S3 Fig).

The R-loop grammar shows overall better prediction capabilities than the existing thermodynamics-based model R-looper [13] for both plasmids and all topologies. When compared to R-looper, our approach reduces the Root Mean Square Deviation (RMSD) by up to 55% and, in most cases it improves the Pearson correlation coefficient by at least 2-fold. Note that the thermodynamics model R-looper [13] is not trained on data. Besides the probability prediction, R-looper also provides an energy landscape for the sequence, which may need to be taken into consideration together with the probability landscape. We refer the reader to the SI Appendix for details. See S6 Table for a comparison against the holdout set and S7 Table for comparison against the full dataset. S8 Table compares the holdout set with the full dataset, and S9 Table gives the same information as S6 Table but for the deterministic symbol assignments.

The grammar rule probabilities vary depending on the plasmid topologies, and thus produce different predictions (Fig 8). Overall the fit to the data is outstanding, with Pearson correlation values from 0.68952 to 0.95165 when compared to the holdout set (S6 Table). The R-loop grammar accurately identifies R-loop clusters along the gene regions in both plasmids and predicts the shift to the left as the supercoiling density increases. As noted in [13], experimental data for hyper-negatively supercoiled pFC8 plasmids present with a much larger number of R-loops near the promoter region as compared with the supercoiled or linear plasmids, where they are largely absent.

## Discussion

The experimental data for hyper-negatively supercoiled pFC8 plasmids show two clusters near the promoter region (Fig 8, blue dotted line, bottom right). The fact that the R-loop grammar predicts one wider cluster near the promoter region can be an artifact of the model.

When processing the experimental data for training, the R-loop grammar assumes that each molecule contains a single R-loop. A small percentage of SMRF-seq reads (9.6% of the data) contains more than one R-loop. In those instances we treat each read as if it were a separate molecule. One could update the grammar to include more than one R-loop per molecule by allowing part *C.* of the grammar rules to have production rules with non-terminal *R*’s on the right-hand side. Such extension is beyond the scope of this paper.

While plasmid topology is an inherent part of the R-looper model [13], the R-looper predictions for hyper-negatively supercoiled plasmids significantly underperform in the detection of R-loops near the transcription start site observed experimentally. Although plasmid topology is not encoded in the R-loop grammar, the model learns the effect of supercoiling from the data and generates predictions that distinguish between plasmid topologies.

In this work we trained the R-loop grammar on a restrictive set of plasmids and topologies, where it performs very well. However, while this is the only SMRF-seq R-loop data available to date, the plasmid sequences for pFC53 and pFC8 are not representative of the much larger set of gene sequences. As more experimental data with a larger array of genomic sequences become available for training, we anticipate that our approach will be an effective universal tool to analyze R-loop formation.

In [27] we showed that the R-loop grammar produces a set of sequences that is regular [21]. Therefore a probabilistic version of this grammar can be described by a Markov chain. This opens the door to a variety of well established techniques (e.g. [28]).

All the code, and the data are available with complete documentation at [23,24].

## Materials and methods

It is known that the initiation of an R-loop is influenced by favorable G-rich DNA sequences [29,30], while sequences spanning the lengths of the R-loops may be less favorable [8,30]. However, any patterns defining possible R-loop termination sequences are currently unknown. To identify preferable DNA sequence patterns that are specific to the initiation, elongation, and termination of R-loops, we carry out an analysis of the experimental results from [13].

### Experimental data

We use experimental R-loop data detected in [13] by SMRF-seq, a method that profiles individual R-loops at ultra-deep coverage [8,18]. The nucleotide sequences of the plasmids pFC53 and pFC8 (previously reported in [13]) share the same backbone and incorporate specific regions known to be prone to R-loop formation [4]. More specifically, pFC53 contains a 1.3-kb portion of the murine Airn CpG island, and pFC8 contains a 942-bp portion of the human SNRPN CpG island. We make the complete SMRF-seq experimental data and software available on GitHub [23] and Zenodo [24]. The template strand $5^{\prime }\;-\;3^{\prime }$ of each plasmid is in FASTA format. The corresponding R-loop locations for each of the three starting plasmid topologies are included in BED files. The data consist of the following: for pFC53 there are 79 co-transcriptional R-loops within the linear, 612 within the supercoiled and 408 within the hyper-negatively supercoiled datasets; for pFC8 there are 116 R-loops within the linear, 104 within the supercoiled and 1044 within the hyper-negatively supercoiled datasets. In total, the data contain 2363 R-loops. Since the gene region in pFC53 is 1749nt long, and that of pFC8 is 1432nt long, the experimental per nucleotide probability of R-loop formation is reported for a total of 3,784,504 nucleotides.

### Training set, holdout set and parameter choice

#### Training and holdout set.

For each topology and each plasmid *P*, we randomly select one-third of the experimental R-loop data as a *holdout set* for later testing. The *P-full training dataset* consists of the remaining two-thirds of the data for *P*. To obtain a *model*, we draw a 10% subsample (without replacement) from each *P*-full training dataset. Hereafter, we refer to this 10% subset as the *P-training set ${𝒯}_{P}$* for the corresponding model. For two plasmids *P*_1_ and *P*_2_ we take the union ${𝒯}_{P_{1}}\cup {𝒯}_{P_{2}}$ of the 10% subsamples to obtain the *union training set* 𝒯, or simply, the *training set*, for a given topology. We assign R-loop grammar symbols to the *k*-mers identified in 𝒯. This assignment is the *dictionary* for 𝒯 (see sections Grammar symbol assignments to the *k*-mers, S2 Text and S2 Fig(a)). We generate a model using the dictionary for 𝒯, i.e. we obtain probabilities for each grammar rule. Next we select distinct training sets to generate an ensemble of models. Finally, we use the average of the ensemble to predict the probability that a given nucleotide falls within an R-loop for any given choice of plasmid and topology (see sections Obtaining a model, S1 Text, and S2 Fig(b)). Figs 7, 8, S4 Fig, S3 Fig and S3 Fig contain the results under various assumptions.

#### Choosing parameters *k* and *p.*

To select the optimal *k* and *p* parameters, we use a nested 3-fold cross-validation on one of the three full training datasets. We reserve one-third of the full training dataset for validation and use the rest of the data for training. We repeat this process for all three non-overlapping validation sets and generate predictions for all pairs (*k*, *p*) where k=3,4,5 and p=7,13. When comparing the model predictions with the experimental validation datasets, the parameters *k* = 4 and *p* = 13 produce the lowest average (over the three validation sets) root mean square deviation (RMSD) and the highest average Pearson correlation coefficient. The results of this analysis are in S3 Table.

### *k*-mer extraction

We consider each plasmid *P* in the $5^{\prime }\;-\;3^{\prime }$ direction of the non-template strand. R-loop locations are specified by their initiation *i* and termination *j* indices, with *j*>*i*. We denote the R-loop segment as the interval of nucleotides [*i*,*j*], i.e. the sequence of nucleotides i,…,j. In order to have each R-loop length as a multiple of *k*, we modify the termination indices of each R-loop as needed (see section S3 Text). The *k*-mer extraction for the dictionary takes place around the initiation and termination sites of an R-loop (Fig 9). This is done separately for each plasmid’s training subset.

Given a *P*-training set ${𝒯}_{P}$ for a given plasmid *P*, we employ a sliding-window approach to extract the *k*-mers specific to the initiation, elongation, and termination of R-loops. Let $p\in {\mathbb{Z}}^{+}$ be a given *padding parameter* and let [*i*,*j*] be an R-loop in ${𝒯}_{P}$. The regions of interest are given by *r*_1_ = *[i*−*k*−*p*,*i*−1*]*, *r*_2_ = *[i*,*i* + *k* + *p*−1*]*, *r*_3_ = *[j*−*k*−*p* + 1,*j]* and *r*_4_ = *[j* + 1,*j* + *k* + *p]*.

We take the *k*-mer [*i*–*k*,*i*–1] containing the *k* nucleotides i−k,…,i−1 before the beginning of the R-loop and shift this window to the left, one nucleotide at a time, for a total of *p* shifts. We perform the shifting as long as the *k*-mer remains in the gene sequence. We discard any extracted *k*-mers that are not fully contained within the gene sequence. The collection of *k*-mers obtained in this way is the set of *k*-mers within region *r*_1_ and is denoted ${ℛ}_{1}$. Similarly, we construct the remaining three collections ${ℛ}_{2}$, ${ℛ}_{3}$, and ℛ_4_ that correspond to *k*-mers within regions *r*_2_, *r*_3_ and *r*_4_, respectively. The set ℛ_1_ consists of the *k*-mers preceding the beginning of an R-loop in ${𝒯}_{P}$, while ${ℛ}_{2}$ (respectively, ${ℛ}_{3}$ and ${ℛ}_{4}$) consists of the *k*-mers at the beginning (respectively, before and after the end) of an R-loop in ${𝒯}_{P}$. Fig 9 illustrates the sliding-window approach.

### Selecting the most relevant *k*-mers

We select the *k*-mers used for the dictionary from the collections ${ℛ}_{i}$, i=1,2,3,4. To each *k*-mer *s* in ${ℛ}_{i}$ we associate a *weight w*_*i*_(*s*) with respect to the region *r*_*i*_ as follows:


$$w_{i}(s)=\frac{n_{i}(s)}{N\cdot m_{s}},$$

where *N* is the number of R-loops in the training set ${𝒯}_{P}$, *n*_*i*_(*s*) is the number of occurrences of *s* in the region *r*_*i*_ across all the R-loops in ${𝒯}_{P}$ (counted with multiplicities), and *m*_*s*_ is the number of occurrences (counted with multiplicity) of *s* within the gene region of the given plasmid. The weight *w*_*i*_(*s*) quantifies the prevalence of *s* in region *r*_*i*_, across all the R-loops in ${𝒯}_{P}$.

The *k*-mers in ${ℛ}_{i}$ are ordered in decreasing order by weight $w_{i}(s_{1})>w_{i}(s_{2})>\ldots$ so that *s*_*i*_ has the ${\text{i}}^{\text{th}}$ highest weight. Table 1 shows a portion of all 115 4-mers in ${ℛ}_{4}$ from the hyper-negatively supercoiled pFC53 R-loop data set.

**Table 1:** Selection of the most relevant 4-mers. Sample in ${ℛ}_{4}$ from the hyper-negatively supercoiled pFC53 R-loop data set. The first column is the ranking *n* of the 4-mers in ${ℛ}_{4}$ after ordering them by weight. The second column lists all the 4-mers where 4-mers with the same ranking are listed in the same field. In this sample the total number of 4-mers in ${ℛ}_{4}$ is 115. The third column indicates the weight $w_{4}(s_{n})$ of *s*_*n*_. The last three columns illustrate the steps of the selection procedure for determining the cutoff point: weight rescaling, entropy calculation, and average entropy computation. The cutoff point–highlighted–is the maximum of the average entropy values.

n	*s*_*n*_	$w_{4}(s_{n})$	$w_{4}^{\prime }(s_{n})$	*H*_*n*_	*h*_*n*_
1	CAAT	0.03571	1.00000	0.00000	0.00000
2	GGAT	0.03214	0.90000	0.04118	0.02059
3	AGGT	0.02976	0.83333	0.06598	0.03572
⋮					
29	GAAG, CCGT, CGCA, ACCA	0.00893	0.25000	0.15051	0.14094
30	CAAG, AAGC, GGTT	0.00824	0.23077	0.14696	0.14147
31	CCCG	0.00794	0.22222	0.14516	blue!250.14168
32	CTCT, TTCA, CGGA, ATTT, TACA, GTGC, GAGT, GCGT, CGTG, GGTA, GTAG, GCCA, AGCC	0.00714	0.20000	0.13979	0.14165
33	AAGG	0.00630	0.17647	0.13294	0.14128
⋮					
40	GCCC	0.00298	0.08333	0.08993	0.13399
41	AAAG	0.00275	0.07692	0.08569	0.13357
42	GCAC	0.00255	0.07143	0.08187	0.13312

In order to identify the most relevant *k*-mers, for each ${ℛ}_{i}$ we determine a cutoff point for thresholding the ordered list using a procedure that relies on entropy reduction [31]. We rescale all *k*-mer weights in ${ℛ}_{i}$ by normalizing with respect to the highest weight (i.e., *s*_1_) with $w_{i}^{\prime }(s_{\ell })=w_{i}(s_{\ell })/w_{i}(s_{1})$. We define the entropy of the *k*-mer $s_{\ell }$ as $H_{\ell }=-w_{i}^{\prime }(s_{\ell })\;\cdot \;\log\left(w_{i}^{\prime }(s_{\ell })\right)$ and compute the average entropy of $s_{1},\ldots ,s_{n}$ as $h_{n}=\frac{1}{n}\sum_{\ell =1}^{n}H_{\ell }$. The (global) maximum of $\left\{h_{1},h_{2},\ldots \right\}$ is set to be the threshold for ${ℛ}_{i}$ [31]. The threshold is achieved when $(n-1)h_{n}\leq h_{1}+\cdots +h_{n-1}$, i.e. when adding a new value *h*_*n*_ is ‘not significant’ with respect to the sum of the already added values.

The threshold reduced list of *k*-mers ${ℛ}_{i}^{*}$, called *highly weighted*, comprises of all *k*-mers $s_{\ell }$ in ${ℛ}_{i}$ such that $h_{\ell }$ is greater than or equal to the threshold value of ${ℛ}_{i}$. Note that there may be several *k*-mers corresponding to the threshold cutoff. In this case, all such *k*-mers are included in the highly weighted list ${ℛ}_{i}^{*}$.

Total number of 4-mers = 115

### Grammar symbol assignments to the *k*-mers

For each plasmid and each set of experimental conditions, we use the list of *k*-mers and their associated weights to assign grammar symbols. This assignment enables us to represent each R-loop with an R-loop grammar word.

**Fig 10:**
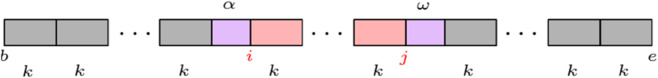
Parsing blocks. Subdivision of the gene region into parsing blocks. We indicate initiation and termination of the R-loop with *i* and *j*, respectively. Each parsing block is a *k*-mer, with the possible exception of the block immediately preceding the R-loop (labeled *α*, in purple) and the block immediately after the R-loop (labeled *ω*, in purple), which could be shorter.

Given an R-loop [*i*,*j*] in ${𝒯}_{P}$ within the gene region [*b*,*e*] of *P*, we focus on three segments [*b*,*i*–1], [*i*,*j*], and [j+1,e] which comprise the sequences preceding, within, and following the R-loop. We subdivide each of the three segments into consecutive and non-overlapping *parsing blocks*. These blocks are *k*-mers, except possibly for the block that ends with *i*–1 and the one that starts with j+1, which could be shorter (i.e., of length <*k*). By construction, the R-loop segment [*i*,*j*] is always a multiple of *k* (see Fig 10 and section S3 Text).

We represent each R-loop in the training set as a word in the R-loop grammar by establishing a correspondence between the parsing blocks within [*b*,*i*–1], [*i*,*j*], and [j+1,e] and the grammar symbols. The correspondence between parsing blocks and grammar symbols is obtained through a symbol assignment function C(ℓ,s) depending on the location ℓ of the first nucleotide of the *k*-mer *s*.

The grammar symbol assignment depends on the parsing block weights generated by the *P*-training set ${𝒯}_{P}$. A parsing block can be highly weighted in a region *r*_*i*_ (for some i=1−4; Fig 6), it can appear in two or more regions but not be highly weighted in any, or not appear in any of the regions. For example, highly weighted parsing blocks in region *r*_4_ are treated as stable DNA:DNA duplexes, and those within region *r*_2_ are treated as stable RNA:DNA duplexes. However, the weighted values can result in ambiguous assignments thus requiring more complex symbol assignment maps. We define the symbol assignment map C(ℓ,s) for highly weighted *k*-mers below and that for not highly weighted blocks in section S2 Text.

#### Highly weighted parsing block assignments.

Let *s* be a *k*-mer in one of the threshold reduced lists ${ℛ}_{i}^{*}$. We set $w_{i}^{*}(s)=0$ if $s\notin {ℛ}_{i}^{*}$. Let $w^{*}(s)=\max\{w_{1}^{*}(s),w_{2}^{*}(s),w_{3}^{*}(s),w_{4}^{*}(s)\}$. Then the assignment map C(ℓ,s) is defined as follows.

- If $w^{*}(s)=w_{i}^{*}(s)=w_{j}^{*}(s)$ with i∈{1,2} and j∈{3,4} then *s* is highly weighted as it appears at both the start and the end of an R-loop. Then
  $$C(\ell ,s)=\left\{ \begin{array}{cc} \delta & \text{ if }\ell \in [b,i-1]\cup [j+1,e]\\ \beta & \text{ if }\ell \in [i,j] \end{array}\right.$$
  treating *s* as both a stable and an unstable DNA:DNA (resp. RNA:DNA) hybrid when the *k*-mer is outside (resp. inside) the R-loop.

Otherwise,

- If $w^{*}(s)\in \{w_{1}^{*}(s),w_{2}^{*}(s)\}$ then C(ℓ,s)=σ for ℓ∈[b,i−1]∪[j+1,e] and $C(\ell ,s)=\hat{\tau }$ for ℓ∈[i,j]. This treats *k*-mers at the beginning of the *R*-loop as stable RNA:DNA hybrids and unstable DNA:DNA duplexes.
- If $w^{*}(s)\in \{w_{3}^{*}(s),w_{4}^{*}(s)\}$ then $C(\ell ,s)=\hat{\sigma }$ for ℓ∈[b,i−1]∪[j+1,e] and C(ℓ,s)=τ for ℓ∈[i,j]. This treats *k*-mers at the end of the *R*-loop as unstable RNA:DNA hybrids and stable DNA:DNA duplexes.

### Dictionary: Grammar symbol assignment for the set 𝒯

Let C(ℓ,s) (respectively, $C^{\left(1\right)}(\ell ,s)$, $C^{\left(2\right)}(\ell ,s)$) denote the symbol assignments for the union training set 𝒯 (respectively, plasmid training sets ${𝒯}_{P_{1}}$, ${𝒯}_{P_{2}}$). The symbol assignment C(ℓ,s) for 𝒯 is based on the symbol assignments $C^{\left(1\right)}(\ell ,s)$ and $C^{\left(2\right)}(\ell ,s)$. If the symbol assignment for both $C^{\left(1\right)}(\ell ,s)$ and $C^{\left(2\right)}(\ell ,s)$ is the same, then $C(\ell ,s)=C^{\left(1\right)}(\ell ,s)$. If the symbol assignments by $C^{\left(1\right)}(\ell ,s)$ and $C^{\left(2\right)}(\ell ,s)$ differ, then we apply two approaches to resolve the conflict - a deterministic one and a stochastic one (see section S2 Text for details).

### R-loop grammar words for 𝒯 to generate a model

To obtain a model for the R-loop grammar, i.e., to specify the probabilities of each of the grammar production rules, we write all R-loops in the union training set 𝒯 as words over the alphabet $\left\{\sigma ,\hat{\sigma },\delta ,\gamma ,\tau ,\hat{\sigma },\beta ,\rho ,\alpha ,\omega \right\}$.

This is done by using the splitting of the gene region into parsing blocks according to the R-loop initiation and termination indices [*i*,*j*] as discussed above, see Fig 10. All but two of the blocks are *k*-mers. To a given *k*-mer, a grammar symbol is assigned according to function C(ℓ,s) for the union training set 𝒯, depending on whether the block precedes, is within, or follows the positions [*i*,*j*]. The lengths of the blocks *α* and *ω* for the transitions can vary between 0 and *k*–1 depending on the values *i*,*j*,*k*. Note that this means that each R-loop word contains exactly one *α* and *ω* even when their corresponding lengths are 0.

### Obtaining a model

A grammar model for the union training set 𝒯 is obtained by assigning probabilities to each of the production rules. Detailed formulas are included in section S1 Text. Using Eq 1 for all words in union training set 𝒯, and then Eq 2, we obtain the final probability for a given nucleotide of a plasmid to be within an R-loop. We produce an ensemble of (in our case 30) models and take the average over all models (S2 Fig).

### Assessing model stability

We use 3-fold cross-validation to assess the stability of model predictions. To do so, we repeat a total of three times the process described under the Training and holdout set subsection, each time selecting a different non-overlapping holdout set and creating an ensemble of models from the respective full training dataset (see S3 Fig).

## Supporting information

S1 Fig Experimental R-loop locations for plasmids pFC53 and pFC8. (PDF)

S2 Fig Schematics of data split, training and prediction for the R-loop grammar model. (PDF)

S3 Fig Predictions from the stochastic R-loop grammar model and from R-looper for different topologies on plasmids pFC8 and pFC53 against the full experimental dataset. (PDF)

S4 Fig Predictions from the deterministic R-loop grammar model and from R-looper for different topologies on plasmids pFC8 and pFC53 against the holdout set. (PDF)

S5 Fig Production rule probabilities. (PDF)

S6 Fig 3-fold predictions from the stochastic R-loop grammar model trained on three distinct two thirds of the data. (PDF)

S1 Table Statistical comparison of production rule probabilities across topologies. (PDF)

S2 Table Spearman and Kendall’s Tau correlations for each production rule. (PDF)

S3 Table Choosing k and *p* parameters. (PDF)

S4 Table Average k-mer coverage of determinate symbols calculated for the union training set dictionaries. (PDF)

S5 TableAverage k-mer coverage of determinate symbols calculated for the union training set of dictionaries for different sets of parameters (k, p).(PDF)

S6 Table RMSD and Pearson correlation coefficient comparison against the holdout set. (PDF)

S7 Table RMSD and Pearson correlation coefficient comparison against the full set. (PDF)

S8 Table Pearson correlation coefficient comparison against the holdout (test) set and the full set (full). (PDF)

S9 Table RMSD and Pearson correlation coefficient comparison with R-looper against the holdout set. (PDF)

S1 Text Training the grammar and generating a model. (PDF)

S2 Text Dictionary: R-loop grammar symbol assignments. (PDF)

S3 Text R-loop data pre-processing. (PDF)
