# Supplementary material for: The R-loop grammar predicts R-loop formation under different topological constraints
Source: PLoS Comput Biol. 2025 Aug 29;21(8):e1013376. doi: 10.1371/journal.pcbi.1013376 (PMC12396753; doi:10.1371/journal.pcbi.1013376)
Supplement: S1 Fig — (PDF) [file pcbi.1013376.s001.pdf]

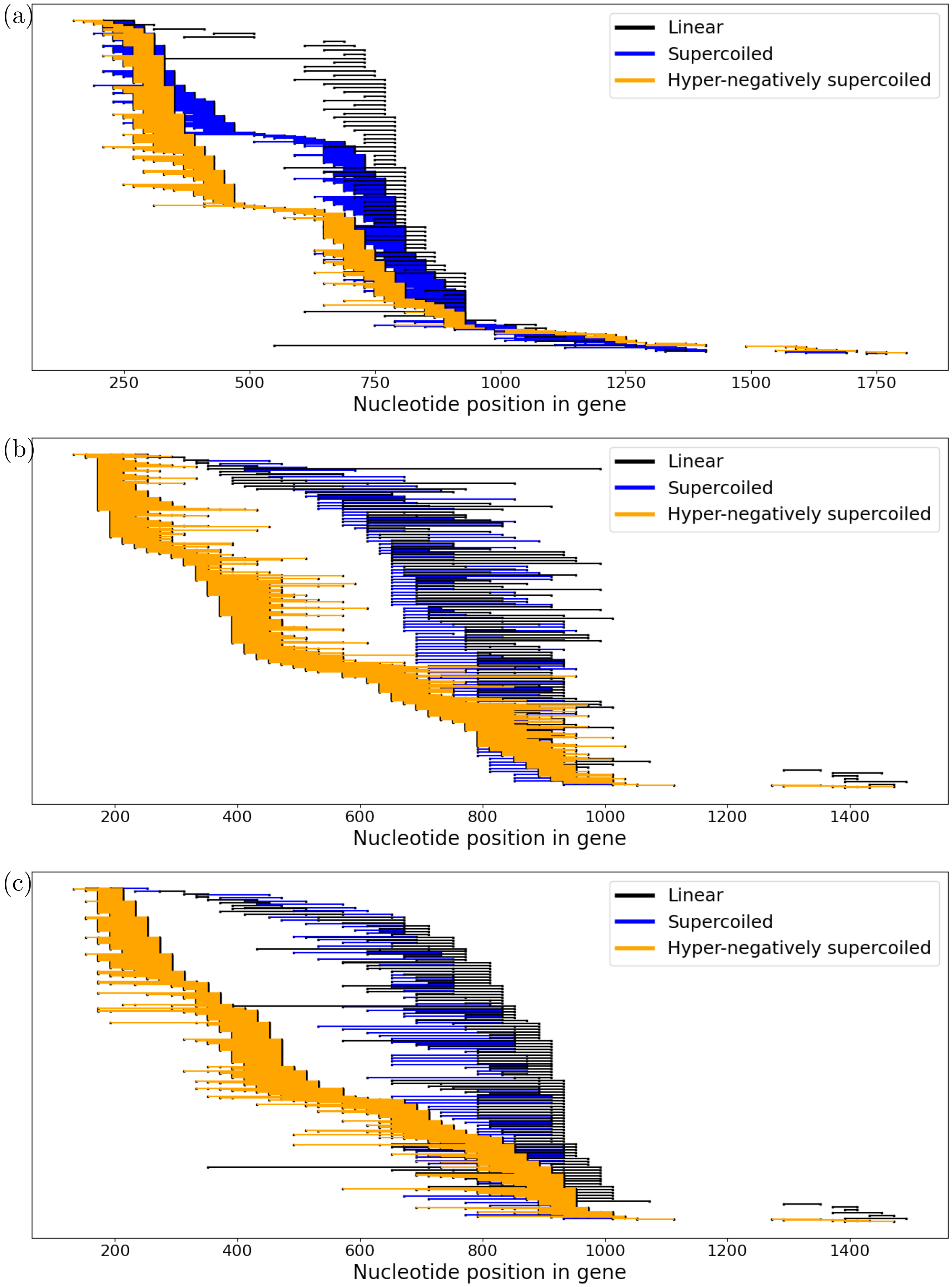

Figure S1. Experimental data. R-loop locations for plasmids pFC53 (a) and pFC8 ((b) and (c)) with starting topology: linear (black); supercoiled (blue); and hyper-negatively supercoiled (orange). The x-axis indicates the nucleotide position of the gene starting at 0, rounded to the nearest 20th nucleotide. Each horizontal line segment corresponds to one experimentally detected R-loop. The R-loops have been sorted by the starting (b) or ending nucleotide ((a) and (c)). Each data set is uniformly spread vertically (116 linear, 104 supercoiled and 1044 hyper-negatively supercoiled for pFC8, and 79 linear, 612 supercoiled and 408 hyper-negatively supercoiled for pFC53). Proportional differences in R-loop initiation under the three conditions can be observed independent of the number of experimental R-loops observed.
