# Supplementary material for: The R-loop grammar predicts R-loop formation under different topological constraints
Source: PLoS Comput Biol. 2025 Aug 29;21(8):e1013376. doi: 10.1371/journal.pcbi.1013376 (PMC12396753; doi:10.1371/journal.pcbi.1013376)
Supplement: S2 Fig — (PDF) [file pcbi.1013376.s002.pdf]

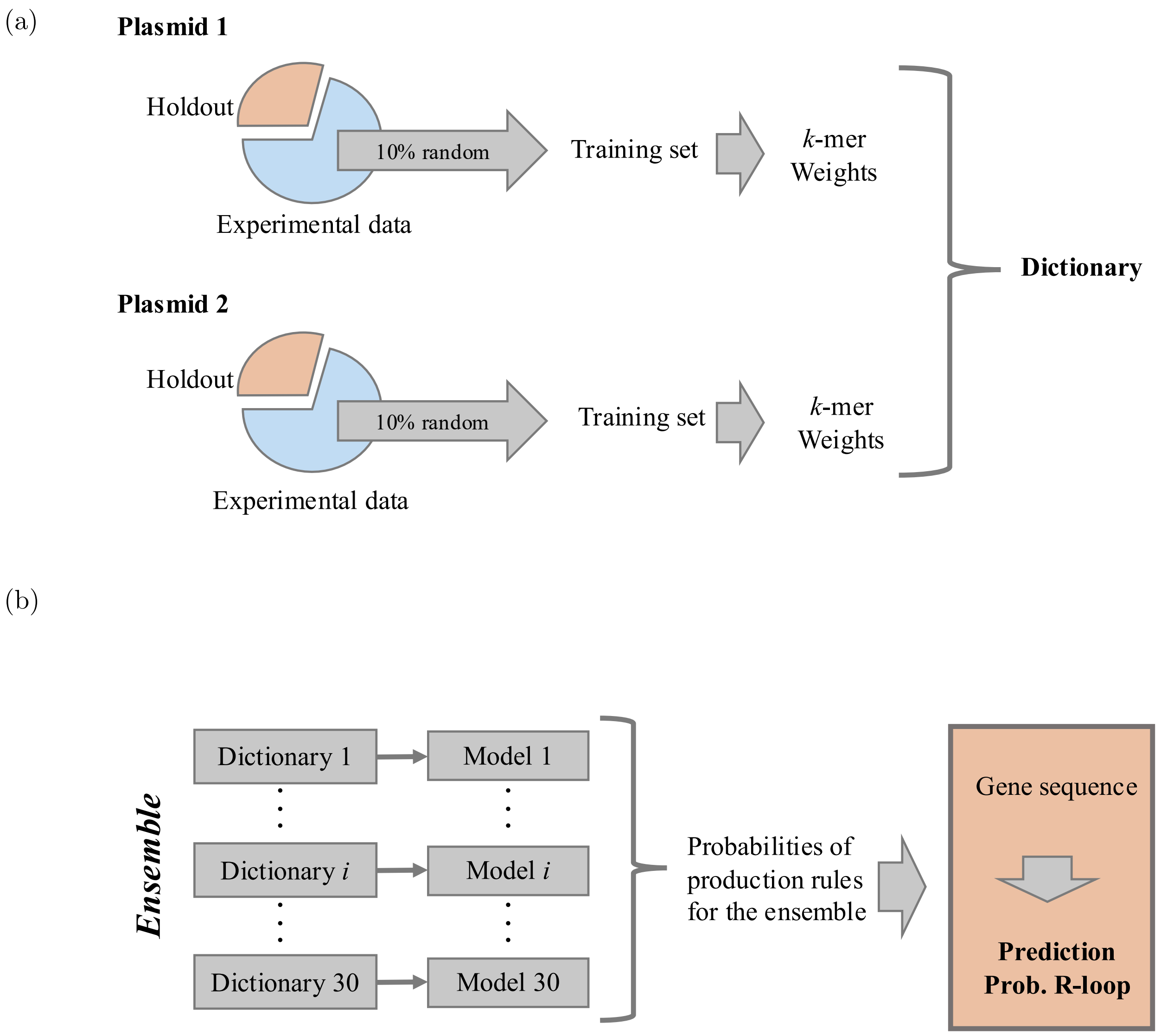

Figure S2. Training and prediction schematic for the R-loop grammar model. (a) Method to define a dictionary trained on two different plasmid datasets. After holding a portion of the data for testing, the training set for the dictionary consists of a random sample of 10% of the remaining data. From each random sample we obtain a different dictionary. (b) Grammar model and prediction. We generate an ensemble of 30 R-loop grammar models that are then used to predict R-loop locations and probabilities on a specific gene sequence.
