# Supplementary material for: The R-loop grammar predicts R-loop formation under different topological constraints
Source: PLoS Comput Biol. 2025 Aug 29;21(8):e1013376. doi: 10.1371/journal.pcbi.1013376 (PMC12396753; doi:10.1371/journal.pcbi.1013376)
Supplement: S3 Fig — (PDF) [file pcbi.1013376.s003.pdf]

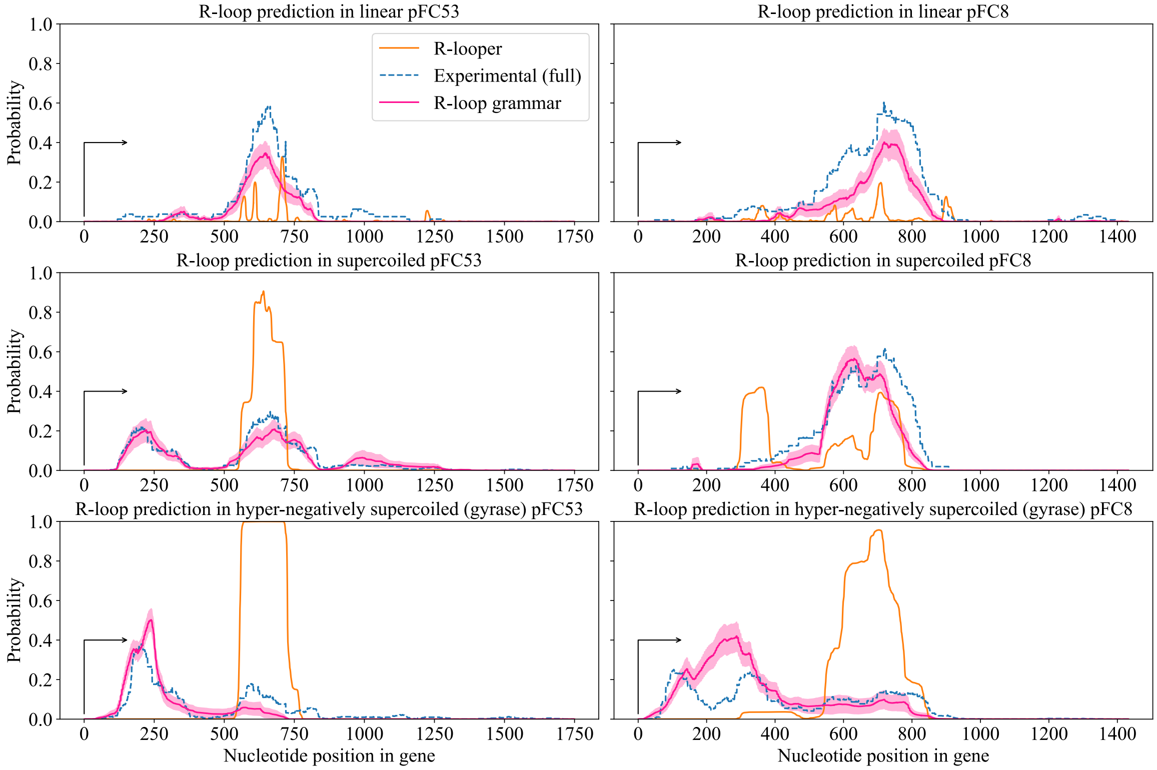

Figure S3. Predictions from the stochastic symbol assignments for the ensemble of R-loop grammar models and from R-looper for different topologies on plasmids pFC8 and pFC53 against the full experimental dataset. The graphs show the predictions from R-looper (orange) and the predictions from the R-loop grammar ensemble of 30 models (pink). The pink shaded area corresponds to the standard error of the mean (s.e.m.) for the ensemble. The dashed blue line shows the observed proportion of R-loops in the full experimental dataset. We indicate the substrate topology in each graph: linear (top row); supercoiled (middle row); hyper-negatively supercoiled (bottom row).
