# Supplementary material for: The R-loop grammar predicts R-loop formation under different topological constraints
Source: PLoS Comput Biol. 2025 Aug 29;21(8):e1013376. doi: 10.1371/journal.pcbi.1013376 (PMC12396753; doi:10.1371/journal.pcbi.1013376)
Supplement: S4 Fig — (PDF) [file pcbi.1013376.s004.pdf]

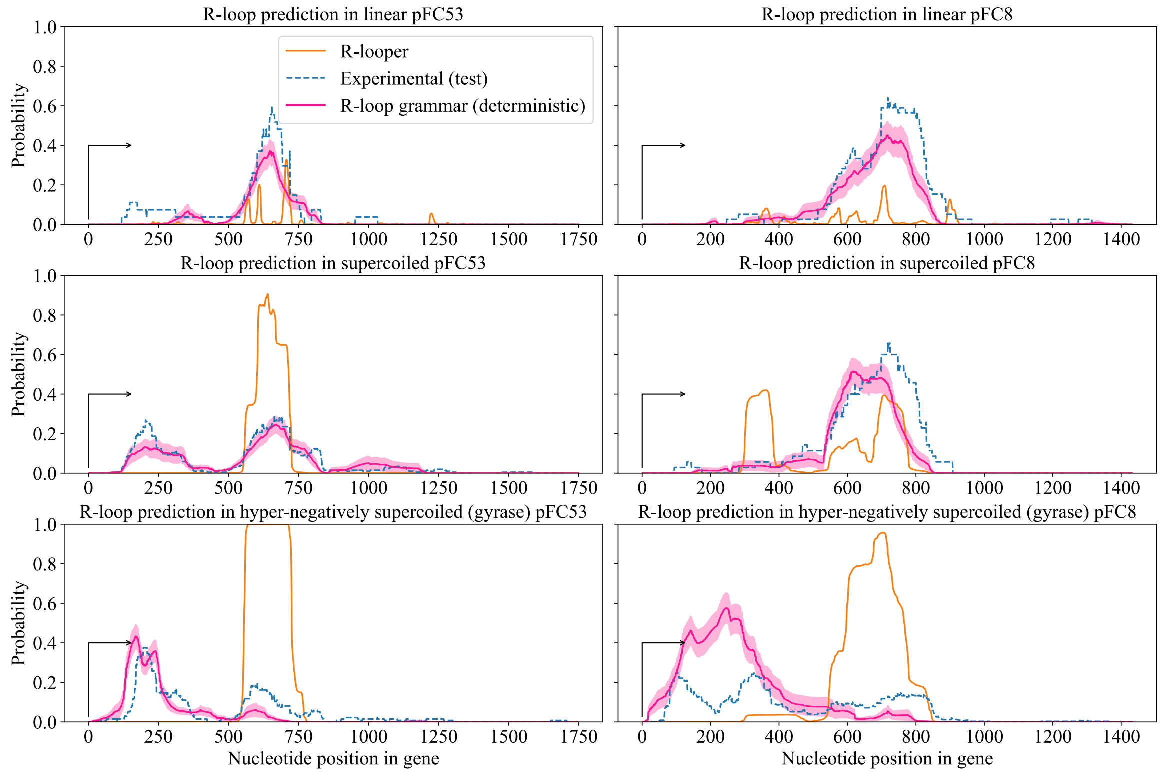

Figure S4. Predictions from the deterministic symbol assignments for the ensemble of R-loop grammar models and from R-looper for different topologies on plasmids pFC8 and pFC53 against the holdout set. The graphs show the predictions from R-looper (orange) and the predictions from the R-loop grammar ensemble of 30 models (pink). The pink shaded area corresponds to the s.e.m. for the ensemble. The dashed blue line shows the observed proportion of R-loops in the holdout set. We indicate the substrate topology in each graph: linear (top row); supercoiled (middle row); hyper-negatively supercoiled (bottom row).
