# Supplementary material for: The R-loop grammar predicts R-loop formation under different topological constraints
Source: PLoS Comput Biol. 2025 Aug 29;21(8):e1013376. doi: 10.1371/journal.pcbi.1013376 (PMC12396753; doi:10.1371/journal.pcbi.1013376)
Supplement: S5 Fig — (PDF) [file pcbi.1013376.s005.pdf]

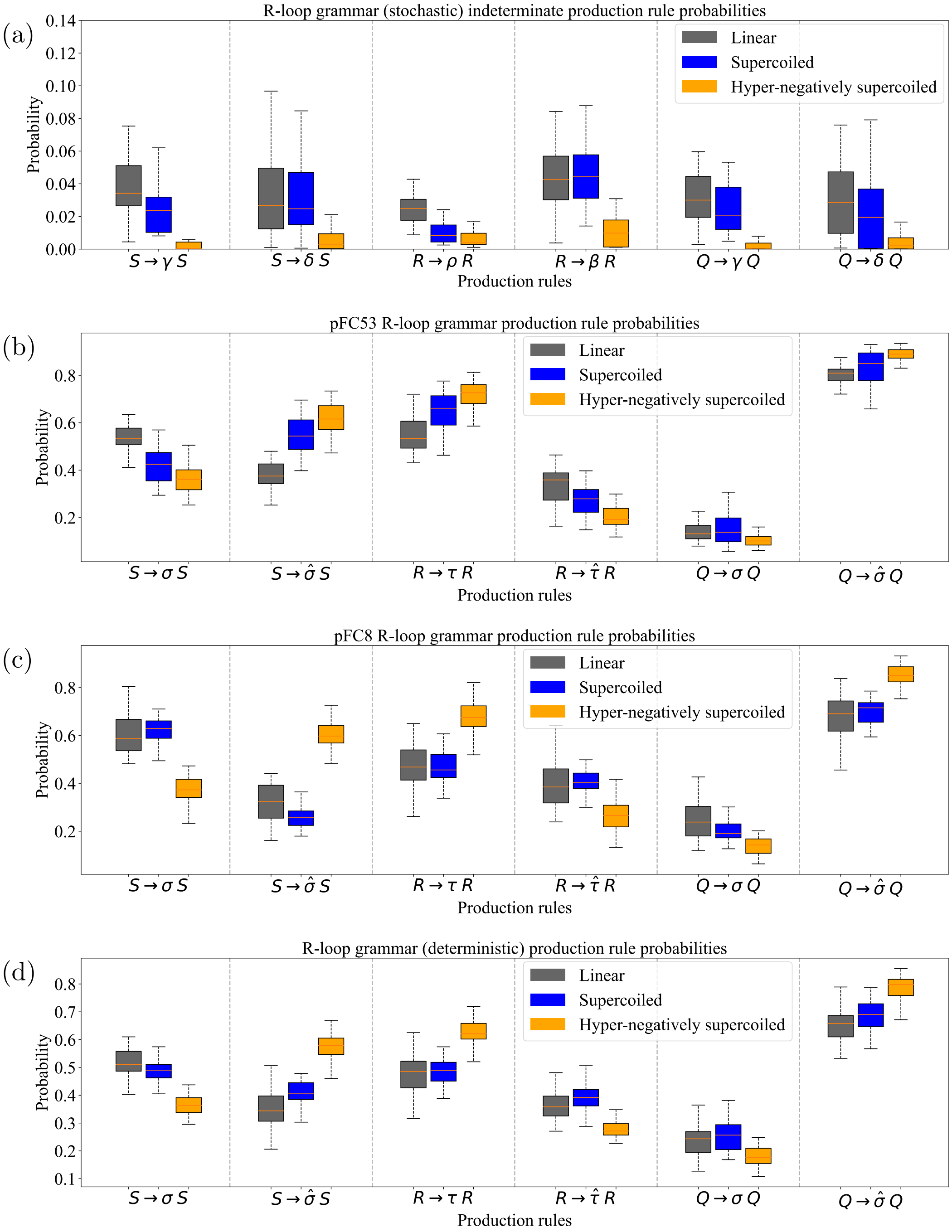

Figure S5. Production rule probabilities. The boxplots illustrate the changes in the probabilities for the six indeterminate production rules (a) and for the main production rules (b-d) related to the stability of the structure before, within, and after an R-loop as the topology from the substrate changes from linear to hyper-negatively supercoiled. In each case, we used the grammar defined with parameters $n = 4$ and $p = 13$. The mid-line of each box is the median, with the first and third quartiles indicated by the box frames. The whiskers represent the largest point not more than 1.5 interquartile range (IQR) beyond the box frame. (a) probabilities obtained with the dictionary for union training sets for the indeterminate rules not shown in Fig. 6. (b) probabilities obtained with dictionary for pFC53-training sets (c) probabilities obtained with dictionary for pFC8-training sets. (d) probabilities for deterministic symbol assignment for union training sets
