# Supplementary material for: The R-loop grammar predicts R-loop formation under different topological constraints
Source: PLoS Comput Biol. 2025 Aug 29;21(8):e1013376. doi: 10.1371/journal.pcbi.1013376 (PMC12396753; doi:10.1371/journal.pcbi.1013376)
Supplement: S6 Fig — (PDF) [file pcbi.1013376.s006.pdf]

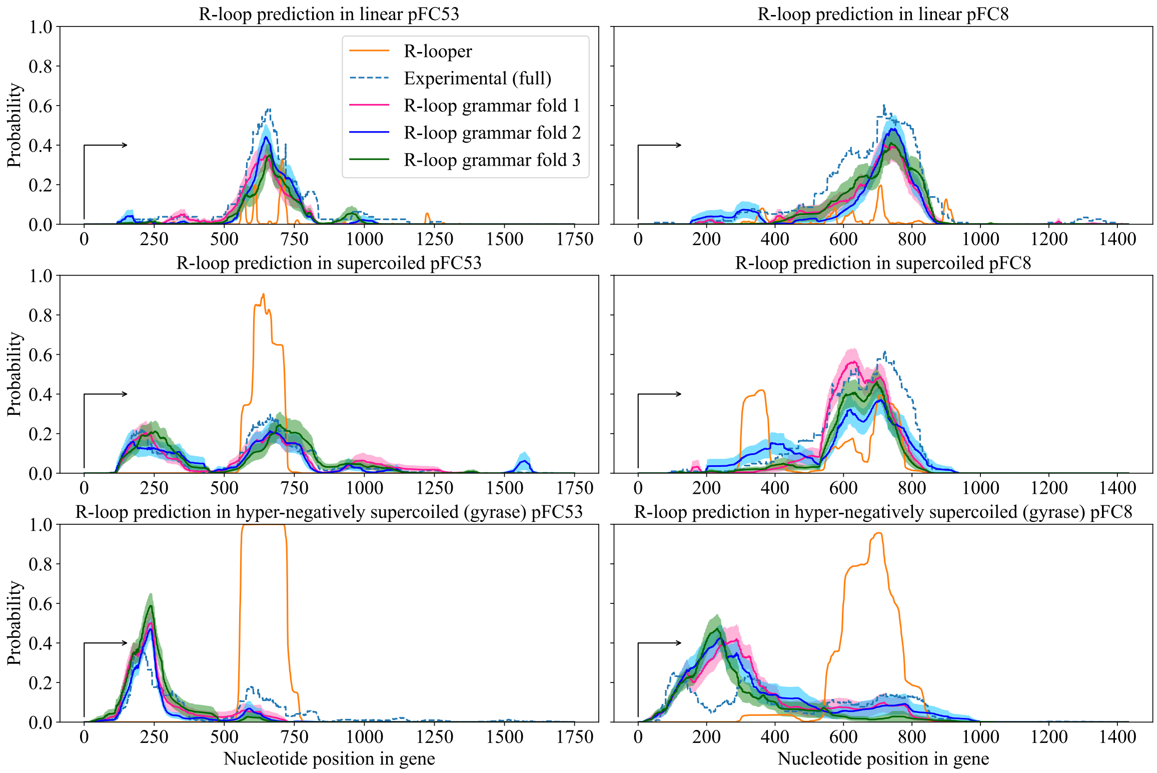

Figure S6. 3-fold predictions from the <u>stochastic</u> R-loop grammar model trained on three distinct two thirds of the data. We computed predictions for different topologies on plasmids pFC8 and pFC53. The graphs show the predictions from each fold from the R-loop grammar ensemble of 30 models (pink, blue, green). The shaded areas correspond to the s.e.m. for each fold ensemble. The orange line corresponds to predictions from R-looper. The dashed blue line shows the observed proportion of R-loops in the full experimental dataset. We indicate the substrate topology in each graph: linear (top row); supercoiled (middle row); hyper-negatively supercoiled (bottom row).
