# Supplementary material for: The R-loop grammar predicts R-loop formation under different topological constraints
Source: PLoS Comput Biol. 2025 Aug 29;21(8):e1013376. doi: 10.1371/journal.pcbi.1013376 (PMC12396753; doi:10.1371/journal.pcbi.1013376)
Supplement: S1 Table — (PDF) [file pcbi.1013376.s007.pdf]

Production Rule	Linear vs. supercoiled	Linear vs. hyper-negatively supercoiled	Supercoiled vs. hyper-negatively supercoiled
$S \rightarrow \sigma S$	0.0788	0.0000	0.0000
$S \rightarrow \hat{\sigma} S$	0.0060	0.0000	0.0000
$R \rightarrow \tau R$	1.0000	0.0000	0.0000
$R \rightarrow \hat{\tau} R$	0.6309	0.0000	0.0000
$Q \rightarrow \sigma Q$	1.0000	0.0000	0.0001
$Q \rightarrow \hat{\sigma} Q$	0.7796	0.0000	0.0000

Table S1. Statistical comparison of production rule probabilities across topologies. After obtaining significant ANOVA results for each production rule, we conducted pairwise t-tests across all topology pairs. We adjusted the p-values for multiple comparisons using the Bonferroni method.
