# Supplementary material for: The R-loop grammar predicts R-loop formation under different topological constraints
Source: PLoS Comput Biol. 2025 Aug 29;21(8):e1013376. doi: 10.1371/journal.pcbi.1013376 (PMC12396753; doi:10.1371/journal.pcbi.1013376)
Supplement: S2 Table — (PDF) [file pcbi.1013376.s008.pdf]

Production Rule	Spearman	p-value	Kendall's Tau	p-value
$S \rightarrow \sigma S$	-0.75	0.0000	-0.61	0.0000
$S \rightarrow \hat{\sigma} S$	0.80	0.0000	0.66	0.0000
$R \rightarrow \tau R$	0.67	0.0000	0.52	0.0000
$R \rightarrow \hat{\tau} R$	-0.53	0.0000	-0.40	0.0000
$Q \rightarrow \sigma Q$	-0.48	0.0000	-0.37	0.0000
$Q \rightarrow \hat{\sigma} Q$	0.65	0.0000	0.51	0.0000

Table S2. Spearman and Kendall's Tau correlations for each production rule. The correlation is computed between the rule probabilities values and the corresponding topologies according to the level of supercoiling.
