# Supplementary material for: The R-loop grammar predicts R-loop formation under different topological constraints
Source: PLoS Comput Biol. 2025 Aug 29;21(8):e1013376. doi: 10.1371/journal.pcbi.1013376 (PMC12396753; doi:10.1371/journal.pcbi.1013376)
Supplement: S3 Table — (PDF) [file pcbi.1013376.s009.pdf]

K	P	Avg. Pearson	(s.e.)	Avg. RMSD	(s.e.)
3	7	0.60641	0.19659	0.13756	0.05114
3	13	0.55434	0.19881	0.16746	0.04238
4	7	0.82141	0.20296	0.08336	0.03897
**4**	**13**	**0.85708**	0.07083	**0.07878**	0.03715
5	7	0.83743	0.07672	0.09311	0.03688
5	13	0.77554	0.08354	0.09567	0.04107

Table S3. Choosing the parameter k and p. Average Pearson correlation coefficient and Root Mean Square Deviation (RMSD) from the two plasmids (pFC53 and pFC8) across topologies. The corresponding standard error of the mean (s.e.) is reported alongside each average. To compute the overall average, we first calculate the mean across the 3-fold cross-validation results for each plasmid, and then average for each k and p pair. The k and p pair that produces the lowest average RMSD and the highest average Pearson correlation coefficient is in bold.
