# Supplementary material for: The R-loop grammar predicts R-loop formation under different topological constraints
Source: PLoS Comput Biol. 2025 Aug 29;21(8):e1013376. doi: 10.1371/journal.pcbi.1013376 (PMC12396753; doi:10.1371/journal.pcbi.1013376)
Supplement: S4 Table — (PDF) [file pcbi.1013376.s010.pdf]

Training on	Topology		
	Linear	Supercoiled	Hyper-negatively supercoiled
pFC8	72.1% (2.4)	69.8% (1.6)	85.0% (1.2)
pFC53	72.4% (2.0)	82.7% (1.9)	83.0% (2.5)
pFC8 $\cup$ pFC53	86.6% (1.5)	91.4% (1.3)	95.2% (0.9)
pFC8 $\cap$ pFC53	31.5% (7.0)	33.1% (7.5)	44.8% (6.6)

Table S4. Average k-mer coverage for determinate symbols (i.e. not γ nor ρ) computed for dictionaries for P-training sets and union training set $\mathcal{T}$ when training the R-loop grammar (with $k = 4$ and $p = 13$) on data from the plasmids pFC8 (row 1), pFC53 (row 2), union (row 3). The overlap of k-mers between two P-training sets is shown with the intersection. For each dictionary, we focused on k-mers that are assigned a symbol that is not one of the indeterminate symbols. There are a total possible $4^4 = 256$ assignments. We computed the average assignment coverage over the ensemble of 30 runs. The results are reported with the Mean% (SD), where SD is the standard deviation.
