# Supplementary material for: The R-loop grammar predicts R-loop formation under different topological constraints
Source: PLoS Comput Biol. 2025 Aug 29;21(8):e1013376. doi: 10.1371/journal.pcbi.1013376 (PMC12396753; doi:10.1371/journal.pcbi.1013376)
Supplement: S5 Table — (PDF) [file pcbi.1013376.s011.pdf]

Topology	$k = 4$		$k = 5$	
	$p = 7$	$p = 13$	$p = 7$	$p = 13$
Linear	84.9% (3.7)	86.6% (1.5)	41.9% (3.3)	43.3% (3.3)
Supercoiled	90.2% (3.8)	91.4% (1.3)	48.9% (1.4)	50.2% (1.3)
Hyper-negatively supercoiled	94.2% (3.0)	95.2% (0.9)	59.1% (1.6)	60.9% (1.8)

Table S5. Average k-mer coverage of determinate symbols calculated for the dictionary for union training sets for different pairs of parameters (k, p). Shown are instances where k-mers are assigned a symbol that is not γ or ρ. We tested the model for $k = 3, 4, 5$ and $p = 7, 13$. When $k = 3$ the symbol assignment exhausts all 64 possible 3-mers and the probabilities of production rules generating γ or ρ are 0. Here we show the average determinate coverage for $k = 4, 5$ and $p = 7, 13$. There are a total possible $4^4 = 256$ assignments for $k = 4$ and $4^5 = 1024$ assignments for $k = 5$, and we look at the average assignment coverage over the ensemble of 30 runs. The results are reported with Mean% (SD), where SD is the standard deviation. The choice $(k, p) = (4, 13)$ provides the largest coverage for the union dictionary across all topologies.
