# Supplementary material for: The R-loop grammar predicts R-loop formation under different topological constraints
Source: PLoS Comput Biol. 2025 Aug 29;21(8):e1013376. doi: 10.1371/journal.pcbi.1013376 (PMC12396753; doi:10.1371/journal.pcbi.1013376)
Supplement: S6 Table — (PDF) [file pcbi.1013376.s012.pdf]

Plasmid	Topology	RMSD		Pearson correlation coefficient	
		R-loop grammar	R-looper	R-loop grammar	R-looper
pFC53	Linear	0.05361	0.11330	0.92021	0.38534
	Supercoiled	0.02754	0.15059	0.94017	0.70360
	Hyper-negatively supercoiled	0.05450	0.28092	0.82407	0.35186
pFC8	Linear	0.09766	0.18832	0.95165	0.39634
	Supercoiled	0.09050	0.15309	0.86910	0.56870
	Hyper-negatively supercoiled	0.08383	0.25526	0.68952	0.25561

Table S6. RMSD and Pearson correlation coefficient calculated by comparing the predictions obtained using the R-loop grammar (dictionary for union training sets; parameters $k = 4$ and $p = 13$) and R-looper against the holdout set.
