# Supplementary material for: The R-loop grammar predicts R-loop formation under different topological constraints
Source: PLoS Comput Biol. 2025 Aug 29;21(8):e1013376. doi: 10.1371/journal.pcbi.1013376 (PMC12396753; doi:10.1371/journal.pcbi.1013376)
Supplement: S7 Table — (PDF) [file pcbi.1013376.s013.pdf]

Plasmid	Topology	RMSD		Pearson correlation coefficient	
		R-loop grammar	R-looper	R-loop grammar	R-looper
pFC53	Linear	0.05935	0.12811	0.97337	0.40471
	Supercoiled	0.02692	0.14704	0.94135	0.74356
	Hyper-negatively supercoiled	0.04861	0.28492	0.86632	0.30082
pFC8	Linear	0.08791	0.17794	0.95792	0.41878
	Supercoiled	0.07429	0.15596	0.92038	0.55947
	Hyper-negatively supercoiled	0.08544	0.25219	0.66803	0.29600

Table S7. RMSD and Pearson correlation coefficient calculated by comparing the predictions obtained using the R-loop grammar (dictionary for union training sets; parameters $k = 4$ and $p = 13$) and R-looper against the full set.
