# Supplementary material for: The R-loop grammar predicts R-loop formation under different topological constraints
Source: PLoS Comput Biol. 2025 Aug 29;21(8):e1013376. doi: 10.1371/journal.pcbi.1013376 (PMC12396753; doi:10.1371/journal.pcbi.1013376)
Supplement: S8 Table — (PDF) [file pcbi.1013376.s014.pdf]

Plasmid	Topology	Pearson correlation coefficient	
		R-loop grammar (test)	R-loop grammar (full)
pFC53	Linear	0.92021	0.97337
	Supercoiled	0.94017	0.94135
	Hyper-negatively supercoiled	0.82407	0.86632
pFC8	Linear	0.95165	0.95792
	Supercoiled	0.86909	0.92038
	Hyper-negatively supercoiled	0.68952	0.66803

Table S8. Pearson correlation coefficient calculated by comparing the predictions obtained using the R-loop grammar (dictionary for union training sets; parameters $k = 4$ and $p = 13$) against the holdout (test) set and the full set (full).
