# Supplementary material for: The R-loop grammar predicts R-loop formation under different topological constraints
Source: PLoS Comput Biol. 2025 Aug 29;21(8):e1013376. doi: 10.1371/journal.pcbi.1013376 (PMC12396753; doi:10.1371/journal.pcbi.1013376)
Supplement: S9 Table — (PDF) [file pcbi.1013376.s015.pdf]

Plasmid	Topology	RMSD		Pearson correlation coefficient	
		R-loop grammar (deterministic)	R-looper	R-loop grammar (deterministic)	R-looper
pFC53	Linear	0.05204	0.11331	0.93690	0.38534
	Supercoiled	0.02876	0.15059	0.93565	0.70360
	Hyper-negatively supercoiled	0.05959	0.28092	0.74841	0.35187
pFC8	Linear	0.07718	0.18832	0.94558	0.39634
	Supercoiled	0.08669	0.15309	0.88208	0.56871
	Hyper-negatively supercoiled	0.13892	0.25526	0.61379	0.25561

Table S9. RMSD and Pearson correlation coefficient calculated by comparing the predictions obtained using the deterministic symbol assignments for the R-loop grammar (dictionary for union training sets; parameters $k = 4$ and $p = 13$) and R-looper against the holdout set.
