# Supplementary material for: The R-loop grammar predicts R-loop formation under different topological constraints
Source: PLoS Comput Biol. 2025 Aug 29;21(8):e1013376. doi: 10.1371/journal.pcbi.1013376 (PMC12396753; doi:10.1371/journal.pcbi.1013376)
Supplement: S1 Text — (PDF) [file pcbi.1013376.s016.pdf]

S1 Text: Training the grammar

Let $\mathcal{D}$ be a dictionary describing grammar symbol associations of k-mers for a union training set $\mathcal{T}$ and let $\mathcal{T} = \mathcal{T}_1 \cup \mathcal{T}_2$ where $\mathcal{T}_1$, $\mathcal{T}_2$ are plasmid training sets for plasmids P_1 and P_2. We associate a probability to each production rule in the grammar, thereby creating a stochastic grammar that models and predicts an R-loop location within a plasmid. The probabilities of the rules with the same non-terminal sum up to 1. Recall that we associate an R-loop grammar word to each R-loop in $\mathcal{T}$ according to the dictionary $\mathcal{D}$.

Each derivation rule probability is determined by the frequency of the rule used in the derivations of the words that correspond to R-loops from a given training set. The grammar is non-ambiguous, i.e., each R-loop word has precisely one parse. Therefore, given a word in the grammar one can easily reverse engineer the sequence of rules that generate the word.

Given a training set of N R-loops, let $\{v_1, v_2, \dots, v_N\}$ be the set of its corresponding grammar words. By construction, for $t \in \{1, \dots, N\}$ each v_t is of the form

$$v_t = v_t^{(1)} x_t \alpha v_t^{(2)} y_t \omega v_t^{(3)} z_t$$

where $x_t, z_t \in \{\sigma, \hat{\sigma}, \gamma, \delta\}$, $y_t \in \{\tau, \hat{\tau}, \rho, \beta\}$, $v_t^{(1)}, v_t^{(3)} \in \{\sigma, \hat{\sigma}, \gamma, \delta\}^*$, $v_t^{(2)} \in \{\tau, \hat{\tau}, \rho, \beta\}^*$. The production rules used to derive the segments $v_t^{(1)} x_t \alpha$ start with the starting non-terminal S.

For a word v, we use the standard notation $|v|$ to indicate the length of v and $|v|_a$ to indicate the number of symbols a appearing in v. For each non-terminal X we denote with $q(X)$ the number of different types of rules of the form $X \to r$ that are in the grammar, where r is any word consisting of terminals and non-terminals. So, for the grammar defined in the main text we have $q(S) = q(R) = q(Q) = 8$. We use Laplace smoothing [1] and add a parameter η to avoid cases with probability 0 (we take $\eta = 1$). We set

$$\mathbb{P}(S \to aS) = \frac{\sum_{t=1}^{N} |v_t^{(1)}|_a + \eta}{\sum_{t=1}^{N} |v_t^{(1)}| + N + \eta q(S)} \quad \text{for } a \in \{\sigma, \hat{\sigma}, \gamma, \delta\},$$

and

$$\mathbb{P}(S \to a\alpha R) = \frac{\sum_{t=1}^{N} |x_t|_a + \eta}{\sum_{t=1}^{N} |v_t^{(1)}| + N + \eta q(S)} \quad \text{for } a \in \{\sigma, \hat{\sigma}, \gamma, \delta\}.$$

We derive the R-loop via productions starting with the non-terminal R, which yield the segments $v_t^{(2)} y_t \omega_{j_t}$. Therefore, we set

$$\mathbb{P}(R \to aR) = \frac{\sum_{t=1}^{N} |v_t^{(2)}|_a + \eta}{\sum_{t=1}^{N} |v_t^{(2)}| + N + \eta q(R)} \quad \text{for } a \in \{\tau, \hat{\tau}, \rho, \beta\},$$

$$\mathbb{P}(R \to a\omega Q) = \frac{\sum_{t=1}^{N} |y_t|_a + \eta}{\sum_{t=1}^{N} |v_t^{(2)}| + N + \eta q(R)} \quad \text{for } a \in \{\tau, \hat{\tau}, \rho, \beta\}.$$

The non-terminal Q is used to derive z_t and the remaining string $v_t^{(3)} z_t$. So we set

$$\mathbb{P}(Q \to aQ) = \frac{\sum_{t=1}^{N} |v_t^{(3)}|_a + \eta}{\sum_{t=1}^{N} |v_t^{(3)}| + N + \eta q(Q)} \quad \text{for } a \in \{\sigma, \hat{\sigma}, \gamma, \delta\},$$

$$\mathbb{P}(Q \to a) = \frac{\sum_{t=1}^{N} |z_t|_a + \eta}{\sum_{t=1}^{N} |v_t^{(3)}| + N + \eta q(Q)} \quad \text{for } a \in \{\sigma, \hat{\sigma}, \gamma, \delta\},$$

Suppose $\mathcal{T}_1$ is a training set with N_1 R-loops for plasmid P_1 and $\mathcal{T}_2$ is a training set with N_2 loops for plasmid P_2. For each of the rules $A \to u$ of the grammar (see sections *Symbol assignment*

and *R-loop production rules* in the main text and here), where A is a non-terminal symbol and u is a word, we compute two probabilities, $\mathbb{P}_{\mathcal{T}_1}(A \to u)$ and $\mathbb{P}_{\mathcal{T}_2}(A \to u)$ with N_1 and N_2 as above, respectively. Finally, we set

$$\mathbb{P}_{\mathcal{T}}(A \to u) = \frac{1}{2}(\mathbb{P}_{\mathcal{T}_1}(A \to u) + \mathbb{P}_{\mathcal{T}_2}(A \to u)).$$

References

[1] Dan Jurafsky and James H. Martin. *Speech and Language Processing: An Introduction to Natural Language Processing, Computational Linguistics, and Speech Recognition*. Pearson Prentice Hall, 2009.

[2] R-loop grammar. `https://github.com/Arsuaga-Vazquez-Lab/R-loopGrammar`, 2023.
