# Supplementary material for: The R-loop grammar predicts R-loop formation under different topological constraints
Source: PLoS Comput Biol. 2025 Aug 29;21(8):e1013376. doi: 10.1371/journal.pcbi.1013376 (PMC12396753; doi:10.1371/journal.pcbi.1013376)
Supplement: S2 Text — (PDF) [file pcbi.1013376.s017.pdf]

S2 Text: Dictionary: R-loop grammar symbol assignments

The *General approach* and the Materials and Methods sections in the main text give an overview of the process for defining the grammar symbols for the highly weighted k-mers. Here we provide details for the symbol assignment of not highly weighted k-mers as well as the list of symbol assignment for the k-mers in the union training set $\mathcal{T}$, which forms the dictionary for the training of the grammar and obtaining a model.

Not highly weighted parsing block assignment. Consider a k-mer s which does not appear in any of the threshold reduced lists $\mathcal{R}_i^*$. We define $w_i(s) = 0$ if $s \notin \mathcal{R}_i$. Let $N_b(s)$, $N_r(s)$ and $N_e(s)$ be the number of times s appears as a parsing block preceding, within and following (resp.) an R-loop in the training set $\mathcal{T}$. Let $M(s) = \max\{N_b(s), N_r(s), N_e(s)\}$.

If the maximum $M(s)$ is achieved in a non-unique way, consider the maximal weight $w(s) = \max\{w_1(s), w_2(s), w_3(s), w_4(s)\}$.

- If $w(s) = 0$, set $C(\ell, s) = \gamma$ for $\ell \in [b, i-1] \cup [j+1, e]$ or $C(\ell, s) = \rho$ for $\ell \in [i, j]$ implying that we cannot determine whether this k-mer is (un)stable DNA:DNA duplex, or RNA:DNA hybrid (resp.).
- If $w(s) \neq 0$ we proceed according to the weights as in the case of highly weighted k-mers.

Otherwise, s has a maximal number of appearances in a unique region. Then we treat the number of occurrences of s within a region as weights, in particular,

- if $M(s) = N_b(s)$ then $C(\ell, s) = \sigma$ for $\ell \in [b, i-1] \cup [j+1, e]$ and $C(\ell, s) = \hat{\tau}$ for $\ell \in [i, j]$;
- if $M(s) = N_e(s)$ then $C(\ell, s) = \hat{\sigma}$ for $\ell \in [b, i-1] \cup [j+1, e]$ and $C(\ell, s) = \tau$ for $\ell \in [i, j]$;
- if $M(s) = N_r(s)$ we focus on the max of the weights $w_2(s)$, $w_3(s)$ in regions r_2, r_3 resp. If $w_2(s) = w_3(s) = 0$ then we pursue as in the case when $M(s)$ is not uniquely defined, otherwise we pursue as in the case of highly weighted k-mers.

k-mer symbols for a union training set. The *General approach* and the Materials and Methods sections in the main text give an overview of the process for defining the grammar symbols for k-mers in the union training set $\mathcal{T}$. Here we give more details.

The assignments obtained after sampling from each plasmid separately have a proportion of indeterminate symbols ranging from 15% to 30.2% (see Table S4). To improve the prediction we incorporate the information from both plasmids by making a symbol assignment to k-mers in the union training set $\mathcal{T}$. The union training set provided a proportion of indeterminate symbols ranging from 4.8% to 13.4% (Table S4). When considering $\mathcal{T}$, the two plasmids may give conflicting information about the symbol assignments. We consider two ways of resolving such conflicting information, stochastic (coin toss) and deterministic, guided by the description of the grammar symbols.

Let $C(\ell, s)$ (respectively, $C^{(1)}(\ell, s)$, $C^{(2)}(\ell, s)$) denote the symbol assignments for the union training set $\mathcal{T}$ (respectively, $\mathcal{T}_{P_1}$, $\mathcal{T}_{P_2}$). If $C^{(1)}(\ell, s) = C^{(2)}(\ell, s)$, we set $C(\ell, s) = C^{(1)}(\ell, s)$. If the symbol assignment conflicts between $C^{(1)}$ and $C^{(2)}$, i.e. $C^{(1)}(\ell, s) \neq C^{(2)}(\ell, s)$, the assignments are resolved by examining the weights of the k-mer s where the the information from the two plasmids disagree, with one set of weights computed from the pFC53 (plasmid $\mathcal{P}_1$) experimental data and the other from pFC8 (plasmid $\mathcal{P}_2$) data. We use the max weight of s within sets $\mathcal{R}_1, \ldots, \mathcal{R}_4$ for both plasmids and use the symbol assignment associated with this weight.

If there are further conflicts, we use one of the deterministic or stochastic ways to resolve the conflicts. On average, in our computations there were 6-8 k-mers with conflicting symbol assignments.

Deterministic assignment of symbols. We resolve conflicts between $C^{(1)}(\ell, s)$ and $C^{(2)}(\ell, s)$ based on the meaning of the grammar symbols as explained in section *Formal grammars and R-loops* in

the main text. More precisely, for a k-mer s and ℓ outside an R-loop, i.e., $\ell \in [b, i-1] \cup [j+1, e]$,

$$C(\ell, s) = \begin{cases} \delta, & \text{if } \{C^{(1)}(\ell, s), C^{(2)}(\ell, s)\} = \{\sigma, \hat{\sigma}\} \\ \hat{\sigma}, & \text{if } \{C^{(1)}(\ell, s), C^{(2)}(\ell, s)\} = \{\hat{\sigma}, \delta\} \\ \sigma, & \text{if } \{C^{(1)}(\ell, s), C^{(2)}(\ell, s)\} = \{\sigma, \delta\} \\ \delta, & \text{if } \{C^{(1)}(\ell, s), C^{(2)}(\ell, s)\} = \{\delta, \gamma\} \\ \hat{\sigma}, & \text{if } \{C^{(1)}(\ell, s), C^{(2)}(\ell, s)\} = \{\hat{\sigma}, \gamma\} \\ \sigma, & \text{if } \{C^{(1)}(\ell, s), C^{(2)}(\ell, s)\} = \{\sigma, \gamma\} \end{cases}$$

We define the assignment to k-mers inside the R-loop, when $\ell \in [i, j]$, by

$$C(\ell, s) = \begin{cases} \beta, & \text{if } \{C^{(1)}(\ell, s), C^{(2)}(\ell, s)\} = \{\tau, \hat{\tau}\} \\ \hat{\tau}, & \text{if } \{C^{(1)}(\ell, s), C^{(2)}(\ell, s)\} = \{\hat{\tau}, \beta\} \\ \tau, & \text{if } \{C^{(1)}(\ell, s), C^{(2)}(\ell, s)\} = \{\tau, \beta\} \\ \beta, & \text{if } \{C^{(1)}(\ell, s), C^{(2)}(\ell, s)\} = \{\beta, \rho\} \\ \hat{\tau}, & \text{if } \{C^{(1)}(\ell, s), C^{(2)}(\ell, s)\} = \{\hat{\tau}, \rho\} \\ \tau, & \text{if } \{C^{(1)}(\ell, s), C^{(2)}(\ell, s)\} = \{\tau, \rho\} \end{cases}$$

Stochastic assignment of symbols. When there is not enough information we perform a random coin toss to resolve the conflict. In the case where $C^{(1)}(\ell, s) \neq C^{(2)}(\ell, s)$ we randomly set $C(\ell, s)$ to be $C^{(1)}(\ell, s)$ or $C^{(2)}(\ell, s)$.
