# Supplementary material for: The R-loop grammar predicts R-loop formation under different topological constraints
Source: PLoS Comput Biol. 2025 Aug 29;21(8):e1013376. doi: 10.1371/journal.pcbi.1013376 (PMC12396753; doi:10.1371/journal.pcbi.1013376)
Supplement: S3 Text — (PDF) [file pcbi.1013376.s018.pdf]

S3 Text: R-loop data pre-processing

We provide all data files and data extraction details in the GitHub repository [2]. The template strand $5' - 3'$ of each plasmid is in FASTA format and the corresponding R-loop locations for each of the three topologies considered are included in BED files. In the following exposition we consider each plasmid in the $5' - 3'$ direction of the non-template strand while R-loop locations are specified by their initiation and termination indices, i and j, with $j > i$. We denote the R-loop segment as interval of nucleotides $[i, j]$, i.e., the sequence of nucleotides $i, \ldots, j$.

We use k-mers ($k \in \mathbb{Z}^+$) to analyze sequence preference for initiation, elongation, and termination of R-loops and assume that the R-loops can be parsed with k-mers. So for each R-loop $[i, j]$, we increase or decrease (whichever modification is smaller) the termination index j to make the R-loop length a multiple of k. For even values of k, if there are two equal modifications of the termination, we choose to slightly elongate the R-loop. Regardless of the parity of k, if the elongation falls outside of the gene region (past the transcription end) then we choose the new termination index to decrease the R-loop size. Figure below shows a schematic representation of this pre-processing of R-loop windows.

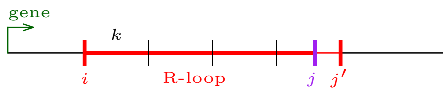

Modification of an R-loop termination index. The experimentally detected R-loop, red thick line, spans the sequence $[i, j]$. Here the new termination index j' increases the termination index j such that the resulting R-loop length is a multiple of k spanning sequence $[i, j']$. Black horizontal line: DNA non-template strand of the plasmid; green arrow: transcription direction.
